# Lipoprotein(a), ABO Blood Types and Clinical Outcomes: Novel Findings and Clinical Implications in Patients With Chronic Coronary Syndrome

**DOI:** 10.1002/mco2.70505

**Published:** 2025-11-28

**Authors:** Hui‐Hui Liu, Chen‐Xi Song, Sha Li, Yan Zhang, Dong Yin, Wei‐Hua Song, Yuan‐Lin Guo, Cheng‐Gang Zhu, Na‐Qiong Wu, Rui‐Xia Xu, Qian Dong, Jie Qian, Yu‐Hui Zhang, Ke‐Fei Dou, Jian‐Jun Li

**Affiliations:** ^1^ Cardiometabolic Center State Key Laboratory of Cardiovascular Disease Fuwai Hospital National Center for Cardiovascular Diseases National Clinical Research Center for Cardiovascular Diseases Chinese Academy of Medical Sciences and Peking Union Medical College Beijing China; ^2^ Heart Failure Center State Key Laboratory of Cardiovascular Disease Fuwai Hospital National Center for Cardiovascular Diseases National Clinical Research Center for Cardiovascular Diseases Chinese Academy of Medical Sciences and Peking Union Medical College Beijing China; ^3^ Center for Coronary Heart Disease State Key Laboratory of Cardiovascular Disease Fuwai Hospital National Center for Cardiovascular Diseases National Clinical Research Center for Cardiovascular Diseases Chinese Academy of Medical Sciences and Peking Union Medical College Beijing China

**Keywords:** blood group, chronic coronary syndrome, lipoprotein(a), outcome

## Abstract

This study aimed to investigate the effect of lipoprotein(a) (Lp(a)) on major adverse cardiovascular events (MACEs) among individuals with chronic coronary syndrome (CCS) according to ABO blood groups. Two independent cohorts of patients with CCS were included consecutively. Blood groups and Lp(a) levels were measured. Patients with the AB group were excluded due to the small sample size. In the exploratory cohort (*n* = 7611), 560 MACEs were recorded over a mean follow‐up of 54.80 months. Stratification analysis revealed that the relationship of elevated Lp(a) levels with prognosis was more pronounced in patients with blood group A or B. Patients with blood group A or B plus medium Lp(a) (HR, 1.93, 95% CI: 1.24–3.01) or high Lp(a) (HR, 2.06, 95% CI: 1.32–3.24) concentrations had a significantly higher risk of MACEs compared to those with blood group O and low Lp(a) levels. Similar results were obtained in the confirmatory cohort (*n* = 7916). In conclusion, our data demonstrated for the first time a more prominent association between Lp(a) and adverse outcomes in CCS patients with non‐O blood group compared to those with blood group O, suggesting that ABO blood group measurement may be clinically useful for decision‐making in Lp(a) intervention.

## Graphical abstract

The present study firstly revealed that the predictive value of Lp(a) for worse outcomes in chronic coronary syndrome patients with A or B blood group was stronger than that in the O group, suggesting that ABO blood group measurement may be clinically helpful for decision‐making in Lp(a) intervention.

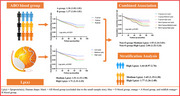

## 1 Introduction

Genetic factors play an important role in the development of cardiovascular diseases (CVDs) and individual variations in response to risk factor modification [1]. As is well known, the ABO blood group is genetically determined by the *ABO* gene [2]. Interestingly, since the identification of ABO blood types, associations between specific ABO blood groups and increased susceptibility to CVDs have been recognized [3, 4, 5, 6]. Numerous studies have suggested that individuals with blood group O have a reduced risk of coronary artery disease (CAD) and acute myocardial infarction (AMI) compared to those with non‐O blood groups [4, 6, 7, 8]. In Gong et al.’s study, non‐O blood group was significantly associated with a 1.49‐fold higher risk of CAD and a 1.24‐fold higher risk of myocardial infarction (MI) compared with blood type O [9]. Meanwhile, a recently published study suggested that blood group A was associated with a relatively higher risk of atherosclerosis in comparison to blood group O, while blood group B was related to an increased risk of MI compared with blood type O [5]. Ketch et al. found that during AMI, patients with non‐O blood types developed a higher thrombus burden than individuals with blood group O, even with less extensive coronary lesions [10]. Moreover, these correlations have been confirmed by subsequent genome‐wide association studies (GWAS) and meta‐analyses [1, 5, 11]. For example, a GWAS by Reilly et al. indicated that among the identified loci, the *ABO* gene was the predominant genetic risk factor for AMI in patients with angiography‐confirmed CAD [12]. Meanwhile, a meta‐analysis revealed a significantly higher risk of MI for non‐O blood groups (OR = 1.25) compared to blood type O [13]. Thus, further study of ABO blood groups may be helpful for guiding more personalized approaches towards the prevention of CVDs in the future.

Previous findings strongly suggest that non‐O blood groups play a significant role in coronary thrombosis, largely attributed to their associated active glycosyltransferase activity [12]. Non‐O blood type‐mediated carbohydrate modification of von Willebrand Factor (VWF) can impair its proteolysis, leading to increased circulating levels of VWF and Factor VIII and promoting thrombogenesis [14]. In contrast, blood group O, characterized by reduced glycosyltransferase activity, is associated with lower levels of VWF and Factor VIII, thereby conferring reduced risk of MI in the context of established CAD [14]. However, the relationship between ABO blood groups and atherosclerotic CVDs may extend beyond thrombotic mechanisms. GWAS has identified *ABO* as a locus influencing the levels of low‐density lipoprotein cholesterol (LDL‐C) [15] and inflammatory biomarkers, such as soluble intercellular adhesion molecule‐1 (ICAM‐1), P‐selectin, and E‐selectin [16]. Thus, non‐O blood groups may further facilitate cardiovascular risk by mediating endothelial dysfunction and inflammation.

Lipoprotein(a) [Lp(a)] is another genetically determined cardiovascular risk factor, causally linked to atherosclerotic CVDs [17, 18, 19, 20]. More than 90% of its plasma concentrations are genetically determined [21, 22]. It's reported that an elevated Lp(a) level is associated with a dramatically increased risk of CAD and MI [18, 23]. Our previous study also indicated that high levels of Lp(a) were significantly related to an elevated risk of recurrent cardiovascular events (CVEs) in patients with established CAD [24]. Lp(a) is characterized by its proatherogenic, prothrombotic, and proinflammatory properties due to its unique structure, a LDL‐like particle including apolipoprotein B100 and apolipoprotein(a) [apo(a)], which contribute to its close association with cardiovascular risk [18, 19].

Nevertheless, the association between Lp(a) and outcomes appears to vary in different situations. For example, the association between elevated Lp(a) levels and CVEs in participants with stable CAD was more prominent in those with hypertension or abnormal glucose metabolism [22, 25]. Post hoc analyses of two large clinical trials showed that the association between high Lp(a) levels and cardiovascular risk was established only when combined with elevated high‐sensitivity C‐reactive protein (hsCRP) levels in both primary and secondary prevention [26, 27]. Additionally, elevated Lp(a) levels and family history of CAD were recently suggested to have independent and additive combined associations with incident CVD [28]. As mentioned above, the coexistence of elevated Lp(a) levels and some established risk factors greatly enhanced cardiovascular risk [22, 25, 26], which may involve certain interactions between them.

Notably, both non‐O blood type and elevated Lp(a) are associated with high inflammatory status and enhanced thrombogenic properties, possibly contributing to their links to CVD risk [9, 29, 30]. Given their common characteristics, we hypothesized that the combined evaluation of plasma Lp(a) levels and ABO blood groups, two genetic risk factors, may better predict CVD risk. Thus, we conducted this study to investigate how ABO blood group modifies the association of Lp(a) with adverse outcomes and to assess the individual and joint prognostic utility of Lp(a) and ABO blood group in two large prospective cohorts of patients with chronic coronary syndrome (CCS).

## 2 Results

### 2.1 Baseline Information

Table 1 provides an overview of the baseline characteristics of the participants in the exploratory cohort. The mean age was 57.5 ± 10.6 years, and 72.4% of them were men. The most common blood group was B (37.1%), closely followed by O (32.2%) and A (30.7%). The median level of Lp(a) was 15.00 mg/dL (interquartile range: 6.69–36.39 mg/dL). Consistent with existing literature, the distribution of Lp(a) concentrations was positively skewed, with a tail extending toward higher values (Figure S1) [22, 31]. Patients with blood group O had the lowest prevalence of prior MI and the lowest levels of hsCRP. Blood group A had the highest levels of total cholesterol and LDL‐C among the three kinds of blood types. However, no significant differences were observed regarding other traditional risk factors, including smoking, diabetes mellitus (DM), and hypertension, across different blood groups.

**TABLE 1:** Baseline characteristics of the exploratory cohort according to ABO blood group.

Variable	Overall (*n *= 7611)	Blood groups^a^			*p* value
		O (*n* = 2453)	A (*n* = 2338)	B (*n *= 2820)	
Age, years	57.5 ± 10.6	57.6 ± 10.6	57.2 ± 10.6	57.6 ± 10.7	0.390
Man, *n* (%)	5509 (72.4)	1780 (72.6)	1690 (72.3)	2039 (72.3)	0.973
BMI, kg/m^2^	25.86 ± 3.18	25.90 ± 3.11	25.82 ± 3.20	25.86 ± 3.21	0.678
Current smokers, *n* (%)	3215 (42.2)	1033 (42.1)	984 (42.1)	1198 (42.5)	0.933
DM, *n* (%)	2110 (27.7)	697 (28.4)	645 (27.6)	768 (27.2)	0.620
Hypertension, *n* (%)	4714 (61.9)	1556 (63.4)	1418 (60.7)	1740 (61.7)	0.132
SBP, mmHg	127 ± 17	127 ± 17	126 ± 17	127 ± 17	0.384
DBP, mmHg	78 ± 11	78 ± 11	77 ± 11	78 ± 11	0.109
Family history of CAD, *n* (%)	1050 (13.8)	351 (14.3)	313 (13.4)	386 (13.7)	0.662
Pre‐MI, *n* (%)	2250 (29.6)	660 (26.9)	741 (31.7)	849 (30.1)	0.002
Pre‐RV, *n* (%)	2187 (28.7)	711 (29.0)	650 (27.8)	826 (29.3)	0.432
LVEF, %	63.48 ± 8.04	63.70 ± 7.63	63.35 ± 8.34	63.41 ± 8.13	0.278
Biochemical parameters					
TC, mmol/L	4.12 ± 1.17	4.11 ± 1.17	4.16 ± 1.15	4.08 ± 1.18	0.053
HDL‐C, mmol/L	1.05 ± 0.29	1.05 ± 0.29	1.05 ± 0.29	1.06 ± 0.29	0.604
LDL‐C, mmol/L	2.49 ± 1.01	2.49 ± 1.05	2.55 ± 0.97	2.45 ± 0.99	0.001
TG, mmol/L	1.49 (1.09–2.08)	1.48 (1.08–2.11)	1.48 (1.09–2.04)	1.49 (1.10–2.10)	0.621
Lp(a), mg/dL	15.00 (6.69–36.39)	14.95 (6.62–38.44)	15.75 (6.96–36.81)	14.48 (6.55–34.28)	0.169
Lp(a) > 50 mg/dL, *n* (%)	1298 (17.1)	423 (17.2)	408 (17.5)	467 (16.6)	0.665
ApoAI, g/L	1.33 ± 0.29	1.33 ± 0.29	1.32 ± 0.31	1.33 ± 0.28	0.263
ApoB, g/L	0.91 ± 0.30	0.91 ± 0.29	0.92 ± 0.29	0.91 ± 0.31	0.098
FPG, mmol/L	5.86 ± 1.76	5.88 ± 1.76	5.84 ± 1.76	5.85 ± 1.76	0.699
HbA1c, %	6.32 ± 1.11	6.33 ± 1.12	6.32 ± 1.07	6.30 ± 1.13	0.580
hsCRP, mg/L	1.36 (0.74–2.82)	1.26 (0.72–2.63)	1.40 (0.73–2.91)	1.40 (0.78–2.86)	0.006
Creatinine, umol/L	78.02 ± 18.47	78.40 ± 18.07	77.47 ± 17.03	78.15 ± 19.91	0.192
Medications at discharge					
Antiplatelet drugs, *n* (%)	7435 (97.7)	2394 (97.6)	2289 (97.9)	2752 (97.6)	0.814
Statins, *n* (%)	7263 (95.4)	2331 (95.0)	2229 (95.3)	2703 (95.9)	0.348
β‐blockers, *n* (%)	5973 (78.5)	1928 (78.6)	1840 (78.7)	2205 (78.2)	0.890
CCB, *n* (%)	2975 (39.1)	954 (38.9)	921 (39.4)	1100 (39.0)	0.935

*Note*: Continuous values are summarized as mean ± SD, median (interquartile range), and categorical variables as percentage. The analysis of variance, nonparametric test, and chi square test were used to compare the differences between groups as appropriate.

Abbreviations: ApoAI, apolipoprotein AI; ApoB, apolipoprotein B; BMI, body mass index; CAD, coronary artery disease; CCB, calcium channel blockers; DBP, diastolic blood pressure; DM, diabetes mellitus; FPG, fasting plasma glucose; HbA1c, glycosylated hemoglobin; HDL‐C, high‐density lipoprotein cholesterol; hsCRP, high‐sensitivity C‐reactive protein; LDL‐C, low‐density lipoprotein cholesterol; Lp(a), lipoprotein(a); LVEF, left ventricular ejection fraction; Pre‐MI, previous myocardial infarction; Pre‐RV, previous revascularization; SBP, systolic blood pressure; TC, total cholesterol; TG, triglyceride.

^a^ Excluded AB blood group due to its small sample size.

Baseline demographic and clinical characteristics of patients in the confirmatory cohort were similar to those in the exploratory cohort, as presented in Table S1.

### 2.2 Individual Association of ABO Blood Group and Lp(a) With the Risk of Major Adverse Cardiovascular Events

Over a mean follow‐up of 54.80 (±18.63) months, a total of 560 Major Adverse Cardiovascular Events (MACEs) were documented in the exploratory cohort, encompassing 221 cardiovascular deaths, 117 nonfatal MIs, and 222 ischemic strokes. As detailed in Table S2, individuals who experienced MACEs were more likely to have blood group A and B, as well as elevated levels of Lp(a), in comparison to those who remained event‐free. Additionally, patients with MACEs were slightly older and had a higher prevalence of DM, hypertension, and prior MI. They also demonstrated higher levels of systolic blood pressure (SBP), glycosylated hemoglobin (HbA1c), creatinine, and hsCRP, and lower levels of diastolic blood pressure (DBP) and left ventricular ejection fraction (LVEF).

**FIGURE 1:**
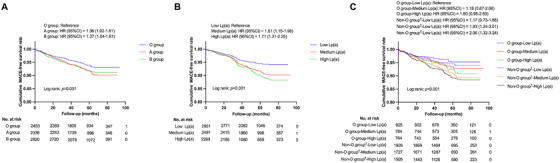
Cumulative MACE‐free survival rate according to ABO blood group and/or Lp(a) levels in the exploratory cohort. (A) ABO blood group^†^; (B) Lp(a) levels; (C) Both ABO blood group^†^ and Lp(a) levels. (MACEs = 560, Total participants = 7611). HR, hazard ratio; Lp(a), lipoprotein(a); MACEs, major adverse cardiovascular events (composite of cardiovascular death, nonfatal myocardial infarction, or ischemic stroke). The significance was examined by the log‐rank tests. HRs were based on Cox regression analyses adjusted for sex, age, body mass index, hypertension, diabetes mellitus, current smoking, prior myocardial infarction, prior revascularization, low‐density lipoprotein cholesterol, triglyceride, high‐sensitivity C‐reactive protein, creatinine, left ventricular ejection fraction, statin use, ABO blood groups, and Lp(a), other than the variables being analyzed. ^†^Excluded AB blood group due to its small sample size.

As shown in Figure S2A, patients with blood group A (16.4, 95% CI: 7.4–21.6) and B (16.0, 95% CI: 11.4–20.6) exhibited a notably elevated incidence of MACEs per 1000 person‐years compared to individuals with blood type O (12.6, 95% CI: 8.2–17.0, both adjusted *p *< 0.05). The groups with medium (16.7, 95% CI: 11.6–21.7) and high Lp(a) levels (18.4, 95% CI: 12.8–23.9) likewise demonstrated a significantly higher incidence of MACEs per 1000 person‐years than the low Lp(a) group (10.9, 95% CI: 7.2–14.6, both adjusted *p* < 0.05; Figure S2B). In addition, Kaplan–Meier analyses revealed that patients with blood group A or B exhibited significantly lower cumulative event‐free survival rates, in contrast to those with blood group O (both *p *< 0.05; Figure 1A). Meanwhile, individuals in the medium‐to‐high Lp(a) groups exhibited lower event‐free survival rates compared to participants in the low Lp(a) group (both *p *< 0.05, Figure 1B). In univariate Cox regression analyses, A and B blood types and medium‐to‐high Lp(a) levels were significantly related to the risk of MACEs. Besides, age, hypertension, DM, prior MI, hsCRP, and creatinine were all positively associated with the occurrence of MACEs, while LVEF was negatively correlated with the risk of MACEs. In further multivariate analysis, A and B blood groups, medium‐to‐high Lp(a) levels, age, hypertension, DM, prior MI, high hsCRP levels, and low LVEF remained significantly associated with the risk of MACEs (Table S3). As presented in Table 2, after adjustment for potential covariates, blood group A had a 1.36‐fold (95% CI: 1.02–1.81) and blood group B had a 1.37‐fold (95% CI: 1.04–1.81) increase in the risk of MACEs, respectively, compared to blood type O. Patients with medium Lp(a) levels had a 1.51 times higher risk of MACEs (95% CI: 1.15–1.98), while those with high Lp(a) levels had a 1.71 times higher risk (95% CI: 1.31–2.25), compared to individuals with low Lp(a) levels. A 1‐SD increase of the log‐transformed Lp(a) [LgLp(a)] was linked to a 21% elevation in the risk of MACEs. The Schoenfeld residuals analyses showed that none of the variables violated the Cox proportional hazards model assumption (ABO blood group: *χ* ^2^ = 2.99, *p* = 0.224; Lp(a) concentration: *χ* ^2^ = 4.97, *p* = 0.084; global test: *χ* ^2^ = 24.96, *p* = 0.126; Table S4).

**TABLE 2:** Cox regression analyses of ABO blood group and Lp(a) levels for predicting MACEs in the exploratory cohort.

Category (events/total subjects)	Crude model HR (95% CI)	Adjusted model HR (95% CI)
ABO blood group^a^		
O group (151/2453)	1.00 (reference)	1.00 (reference)
A group (188/2338)	1.36 (1.04–1.78)^*^	1.36 (1.02–1.81)^*^
B group (221/2820)	1.33 (1.02–1.73)^*^	1.37 (1.04–1.81)^*^
Lp(a)		
Low Lp(a) (149/2851)	1.00 (reference)	1.00 (reference)
Medium Lp(a) (207/2491)	1.55 (1.20–2.00)^*^	1.51 (1.15–1.98)^*^
High Lp(a) (204/2269)	1.74 (1.34–2.28)	1.71 (1.31–2.25)^**^
Per 1‐SD increase of LgLp(a)	1.23 (1.11–1.37)^**^	1.21 (1.08–1.36)^*^
ABO blood group^a^ and Lp(a)		
O group‐Low Lp(a) (44/925)	1.00 (reference)	1.00 (reference)
O group‐Medium Lp(a) (50/764)	1.35 (0.80–2.28)	1.18 (0.67–2.06)
O group‐High Lp(a) (57/764)	1.70 (1.03–2.82)^*^	1.60 (0.95–2.69)
Non‐O group‐Low Lp(a) (105/1926)	1.24 (0.79–1.95)	1.17 (0.73–1.86)
Non‐O group‐Medium Lp(a) (157/1727)	2.00 (1.30–3.08)^*^	1.93 (1.24–3.01)^*^
Non‐O group‐High Lp(a) (147/1505)	2.18 (1.41–3.37)^**^	2.06 (1.32–3.24)^*^

*Note*: Adjusted model adjusted for sex, age, body mass index, hypertension, diabetes mellitus, current smoking, prior myocardial infarction, prior revascularization, low‐density lipoprotein cholesterol, triglyceride, high‐sensitivity C‐reactive protein, creatinine, left ventricular ejection fraction, statin use, ABO blood groups, and Lp(a), other than the variables being analyzed. The significance was tested by univariate and multivariate Cox regression analyses.

Abbreviations: CI, confidence interval; HR, hazard ratio; LgLp(a), log‐transformed Lp(a); Lp(a), lipoprotein(a); MACEs, major adverse cardiovascular events.

^a^ Excluded AB blood group due to its small sample size.

* *p *< 0.05.

** *p *< 0.001.

When MACEs were considered separately, we observed that blood group A had a significant association with ischemic stroke (HR, 1.64, 95% CI, 1.02–2.66), a nominal association with cardiovascular death (HR, 1.43, 95% CI, 0.92–2.23), and a neutral relationship with nonfatal MI (HR, 0.82, 95% CI, 0.43–1.55), while blood group B was linked to a nonsignificant increase in the risk of cardiovascular death (HR, 1.29, 95% CI, 0.83–1.97), nonfatal MI (HR, 1.24, 95% CI, 0.71–2.15), and ischemic stroke (HR, 1.57, 95% CI, 0.99–2.51). In addition, patients with high Lp(a) concentrations were found to be at greater risk of cardiovascular mortality (HR, 1.68, 95% CI, 1.08–2.62), nonfatal MI (HR, 1.63, 95% CI, 0.91–2.95), and ischemic stroke (HR, 1.65, 95% CI, 1.05–2.60; Table S5).

**FIGURE 2:**
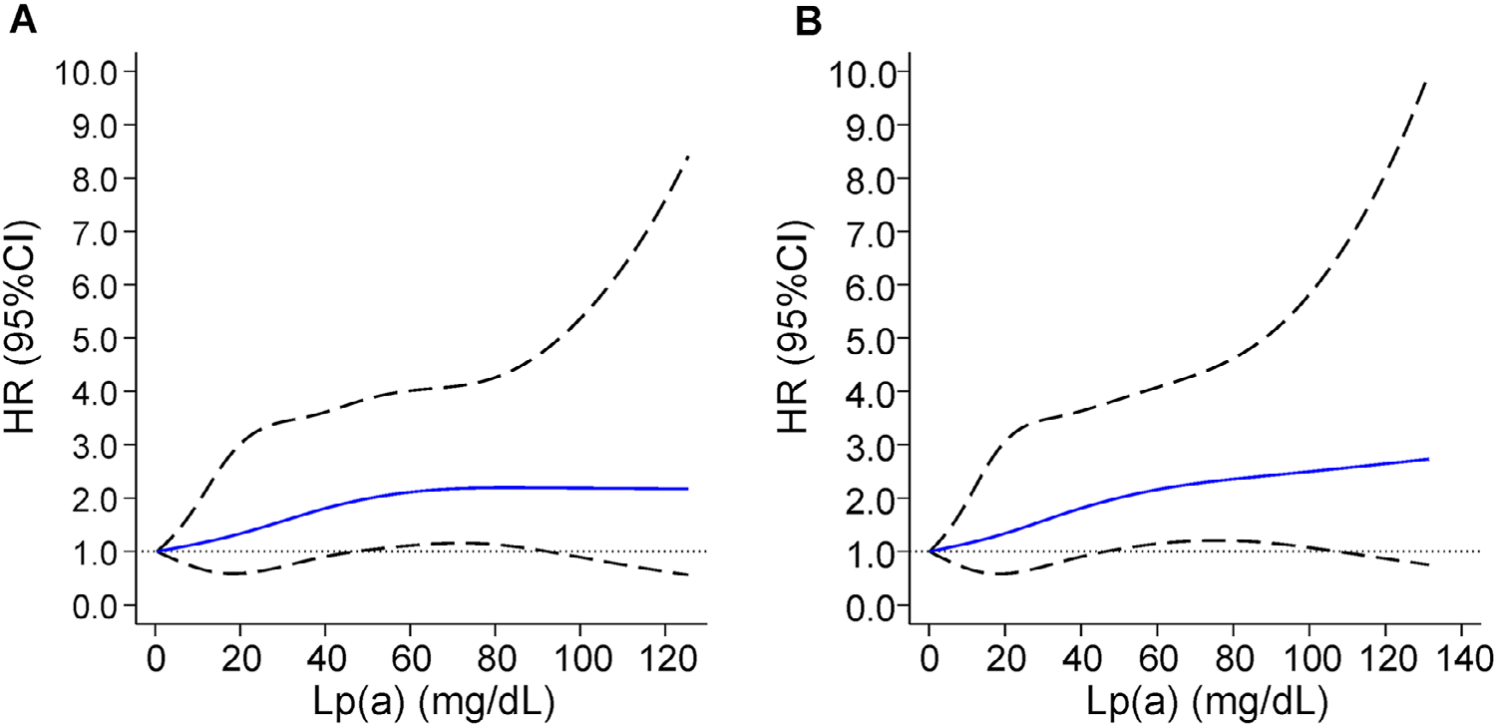
Sex‐ and age‐adjusted RCS plot of Lp(a) and risk of MACEs according to ABO blood group among patients in the exploratory cohort (A) O blood group; (B) A or B blood group. (Events = 560, Total participants = 7611). HR, hazard ratio; Lp(a), lipoprotein(a); RCS, restricted cubic spline; MACEs, major adverse cardiovascular events.

In stratification analysis according to ABO blood groups, data showed that the relationship between medium‐to‐high concentrations of Lp(a) and MACEs in participants with non‐O blood groups was statistically significant, while the notable correlation between high Lp(a) levels and MACEs was attenuated after adjusting for potential covariates in those with blood type O (*p* for interaction = 0.015; Table 3), so did the associations between Lp(a) levels and each component of MACEs (Table S6). A statistically significant synergistic relationship was observed between ABO blood group and Lp(a) [relative excess risk of interaction (RERI): 0.65, 95% CI, 0.03–1.28; attributable proportion (AP): 0.27, 95% CI, 0.01–0.56]. Restricted cubic spline (RCS) analysis revealed a nonlinear, positive correlation between Lp(a) levels and MACEs with a steeper slope in individuals with non‐O blood type as opposed to those with blood type O (Figure 2).

**TABLE 3:** Subgroup analyses of the association between Lp(a) and MACEs according to ABO blood group in the exploratory cohort.

ABO blood group^a^ (events/total subjects)	Lp(a) categories (events/total subjects)	Crude model HR (95% CI)	Adjusted model HR (95% CI)
O group (151/2453)	Low Lp(a) (44/925)	1.00 (reference)	1.00 (reference)
	Medium Lp(a) (50/764)	1.34 (0.80–2.27)	1.20 (0.68–2.10)
	High Lp(a) (57/764)	1.71 (1.03–2.82)^*^	1.64 (0.97–2.76)
	Per 1‐SD increase of LgLp(a)	1.30 (1.05–1.61)^*^	1.23 (0.98–1.54)
Non‐O group^a^ (409/5158)	Low Lp(a) (105/1926)	1.00 (reference)	1.00 (reference)
	Medium Lp(a) (157/1727)	1.62 (1.19–2.21)^*^	1.65 (1.19–2.30)^*^
	High Lp(a) (147/1505)	1.76 (1.29–2.41)^**^	1.77 (1.26–2.48)^*^
	Per 1‐SD increase of LgLp(a)	1.21 (1.07–1.37)^*^	1.20 (1.05–1.38)^*^

*Note*: Adjusted model adjusted for age, sex, body mass index, hypertension, diabetes mellitus, current smoking, prior myocardial infarction, prior revascularization, low‐density lipoprotein cholesterol, triglyceride, high‐sensitivity C‐reactive protein, creatinine, left ventricular ejection fraction, and statin use. The significance was tested by univariate and multivariate Cox regression analyses.

Abbreviations: CI, confidence interval; HR, hazard ratio; LgLp(a), log‐transformed Lp(a); Lp(a), lipoprotein(a); MACEs, major adverse cardiovascular events.

^a^ Excluded AB blood group due to its small sample size.

* *p *< 0.05.

** *p *< 0.001.

In the confirmatory cohort, a comparable pattern of associations of ABO blood group and Lp(a) levels with the risk of MACEs was discerned, in which 254 MACEs (105 cardiovascular deaths, 94 nonfatal MIs, and 55 ischemic strokes) were recorded over a mean follow‐up of 45.73 ± 12.55 months. The comparisons of baseline characteristics of individuals with and without MACEs were detailed in Table S7, which were similar to the exploratory cohort. Patients with blood type A and B had significantly higher absolute risk of MACEs compared to participants with blood group O, as well as the comparison of patients in medium and high Lp(a) groups with those in low Lp(a) group (all adjusted *p *< 0.05; Figure S2A,B). As shown in Figure S3A, the Kaplan–Meier survival curves exhibited that participants with A and B blood groups had significantly higher risk of MACEs compared to those with blood group O. After adjusting for potential covariates, blood type A and B remained significantly associated with a higher risk of MACEs compared with blood group O. Additionally, patients with medium and high Lp(a) levels had significantly higher relative risk of MACEs than those with low Lp(a) levels and a 1‐SD increment of LgLp(a) levels was related to a 21% elevation in the risk of MACEs (Figure S3B and Table S8). The Cox proportional hazards model assumption was also validated by the Schoenfeld residuals analyses (ABO blood group: *χ* ^2^ = 0.11, *p* = 0.944; Lp(a): *χ* ^2^ = 3.14, *p* = 0.208; global test: *χ* ^2^ = 21.14, *p* = 0.132). Further stratification analysis showed that the significant relationship between elevated Lp(a) and MACEs risk was more prominent in patients with A or B blood group compared to those with blood type O (*p* for interaction = 0.047; Table S9). Similarly to the exploratory cohort, a statistically significant synergistic association was observed between non‐O blood group and high levels of Lp(a) and the risk of MACEs (RERI: 0.86, 95% CI, 0.03–1.68; AP: 0.31, 95% CI, 0.02–0.64). Regarding the components of MACEs, we observed that blood group A and B were significantly related to the risk of cardiovascular death, while medium to high levels of Lp(a) had a significant association with the risk of stroke (Table S10). Subgroup analysis indicated that the significant correlation between elevated Lp(a) levels and ischemic stroke mainly existed in patients with non‐O blood group (Table S11). The RCS plot curves of Lp(a) and MACEs according to ABO blood groups among patients in the confirmatory cohort were similar to those in the exploratory cohort (Figure S4).

### 2.3 Joint Association of ABO Blood Group and Lp(a) With the Risk of MACEs

When categorizing all individuals in the exploratory cohort according to ABO blood groups and Lp(a) levels together, the corresponding MACEs per 1000 person‐years were 9.8 (95% CI: 3.5–16.0), 13.0 (95% CI: 5.0–21.1), 15.6 (95% CI: 6.8–24.4), 11.4 (95% CI: 6.7–16.1), 18.3 (12.0–24.6), and 19.7 (95% CI: 12.6–26.8), respectively, from Groups 1 to 6.

Compared to O group‐Low Lp(a) group, both Non‐O group‐Medium Lp(a) and Non‐O group‐High Lp(a) groups exhibited a significantly elevated risk of MACEs (both adjusted *p *< 0.05; Figure S2C). The Kaplan–Meier analyses showed that the cumulative event‐free survival rates were notably lower for participants in O blood group with high Lp(a) levels group or non‐O blood group plus medium/high Lp(a) levels groups when compared to those in the reference group (O group‐Low Lp(a), all *p *< 0.05; Figure 1C). In multivariate Cox proportional hazards model analysis, Non‐O group‐Medium Lp(a) and Non‐O group‐High Lp(a) groups had a 1.93‐fold (95% CI: 1.24–3.01) and 2.06‐fold (95% CI: 1.32–3.24) increased risk of MACEs, respectively, in comparison to the reference group. In contrast, the remaining three groups did not show a statistically significant increase in the risk of MACEs (Table 2). In individual analysis of MACEs, a comparable relationship was identified between the combination of ABO blood groups and Lp(a) and cardiovascular death, nonfatal MI, and ischemic stroke separately (Table S5).

The analysis of the confirmatory cohort showed that participants in Non‐O group‐Medium Lp(a) (10.6 MACEs per 1000 person‐years) and Non‐O group‐High Lp(a) (12.2 MACEs per 1000 person‐years) groups had a significant higher absolute risk of MACEs, compared to patients in the O group‐Low Lp(a) group (4.9 MACEs per 1000 person‐years) (both adjusted *p *< 0.05; Figure S2C). Moreover, as shown in Figure S3C, among six groups classified based on blood types and Lp(a) levels, Non‐O group‐High Lp(a) group exhibited the lowest cumulative event‐free survival rate, followed by Non‐O group‐Medium Lp(a) group. In further multivariate Cox regression analysis, individuals with non‐O blood groups accompanied by medium/high Lp(a) levels continued to show a markedly increased risk of MACEs compared to the reference group (Table S8). The risk of each component of MACEs across the six groups presented a similar trend with the composite outcome (Table S10).

As shown in Table S12, the C‐statistic analysis indicated that the addition of the combination of ABO blood group and Lp(a) to the SCORE2 risk model significantly increased the C‐statistic value in both the exploratory (ΔC‐statistic: 0.032, 95% CI, 0.004–0.057; *p* = 0.020) and confirmatory (ΔC‐statistic: 0.023, 95% CI, −0.002 to 0.040; *p* = 0.030) cohorts. Nevertheless, there was no significant changes in the predictive efficacy by adding blood type or Lp(a) alone to the SCORE2 model.

### 2.4 Subgroup and Sensitivity Analyses of the Association of ABO Blood Group and Lp(a) With the Risk of MACEs

In both cohorts of patients with CCS, subgroup analyses according to sex showed a generally consistent trend of the individual and joint associations of ABO blood group and Lp(a) concentrations with the risk of MACEs in men and women (Tables S13 and S14). Baseline characteristics of participants with blood group AB are detailed in Table S15. Sensitivity analysis by incorporating the AB blood group further demonstrated a stronger association between elevated Lp(a) levels and MACEs in non‐O blood groups (Tables S16 and S17) and similar patterns of the combined association of ABO blood group and Lp(a) with the risk of MACEs (Tables S18 and S19).

## 3 Discussion

In the present study, with two large prospective cohorts of patients with CCS, we observed for the first time that both non‐O blood groups and elevated Lp(a) levels were independently and significantly correlated with an increased risk of MACEs, while the association of Lp(a) with MACEs risk was more pronounced in patients with blood group A or B compared to those with blood group O. Of note, patients with the coexistence of elevated Lp(a) and non‐O blood groups were at the highest risk of MACEs among subgroups categorized according to both ABO blood type and Lp(a). The interaction analysis showed a synergistic relationship between non‐O blood group and elevated Lp(a) levels for their associations with MACEs. Our novel findings may be of certain clinical importance for more accurate risk‐stratification and for guiding clinical decision‐making.

According to current knowledge, much of the hereditary aggregation of CAD could be attributed to genetic risk factors [1]. *ABO* is identified as the most significant locus in a GWAS of venous thromboembolism [12]. The top single nucleotide polymorphisms for venous thromboembolism in the *ABO* gene are strongly associated with MI in patients with angiography‐confirmed CAD [12]. The *ABO* gene encodes proteins (transferase A, a 1‐3‐*N*‐acetylgalactosaminyltransferase; transferase B, a 1‐3‐galactosyltransferase) related to the ABO blood group system [5, 6], which has been suggested to play an important role in elevating the risk of CVDs through complex mechanisms [5, 32, 33]. Individuals with non‐O blood groups exhibited an elevated risk of atherosclerosis, CAD, acute coronary syndrome (ACS), and MI in comparison to blood type O [5, 7, 32]. A study by Carpeggiani indicated that non‐O blood groups were linked to a 24% increased risk of mortality in patients with ischemic heart disease, and MI patients had a higher proportion of A and B blood groups [3]. Recently, with the large UK biobank cohort, Groot et al. also demonstrated that ABO blood group was significantly related to cardiovascular outcomes, in which blood group A and B were associated with a 56% increased risk of thromboembolic events compared to blood group O [5]. In most previous studies, O blood group exhibited a “possible protective” role against cardiovascular risk. However, this phenomenon has not been transferred to clinical guidelines, which may require more persuasive evidence. Consistently, our results also showed a similarly increased risk of MACEs in patients with A and B blood groups compared to individuals with blood group O, with an absolute risk increase of 3.8 per 1000 person‐years and 3.4 per 1000 person‐years respectively. Therefore, we pooling blood group A and B together in subsequent analyses.

Plasma Lp(a) concentrations, which are largely determined by hereditary factors, have been treated as a major residual risk factor for CVDs [28]. An increasing body of evidence suggests that Lp(a) participates in the pathogenesis and development of CVDs [34, 35]. Moreover, it has been demonstrated that higher levels of Lp(a) are correlated with an elevated risk of CVEs across diverse populations, including those with premature or stable CAD, ACS, familial hypercholesterolemia, DM, and so forth [22, 24, 36, 37, 38]. Antisense oligonucleotides (ASOs) and small interfering RNAs (siRNAs) are the primary RNA‐based therapeutic strategies being investigated to reduce plasma Lp(a) levels. Both classes of drugs lower Lp(a) by targeting destruction of the *LPA* mRNA, thereby reducing the synthesis of the apo(a) protein essential for Lp(a) assembly [39]. A recently published Phase II randomized controlled trial (RCT) of Lepodisiran, a long‐duration siRNA targeting Lp(a), showed that it significantly decreased plasma Lp(a) levels within 60–180 days after administration [40]. Lp(a) HORIZON, an ongoing phase III RCT, will determine the effect of pelacarsen (an ASO) on the incidence of MACEs among patients with elevated Lp(a) concentrations and established CVD. Most recently, another Phase III trial of Olpasiran (a siRNA) called OCEAN(a) started enrollment as well [39]. It's optimistic that these Phase III clinical trials will demonstrate positive results, ultimately benefiting individuals with CVD combined with elevated Lp(a) concentrations. However, the prognostic value of circulating Lp(a) levels for cardiovascular risk may vary among different individuals [22, 25, 26, 27, 41]. In line with prior studies, our data showed that elevated Lp(a) level was an independent risk factor for MACEs among patients with established CCS. Meanwhile, we firstly observed that the significant association between elevated Lp(a) levels and MACEs risk was more prominent in patients with non‐O blood group. Besides the patients with O or non‐O blood group combined with high Lp(a) levels, individuals with non‐O blood group and medium levels of Lp(a) should also receive intensified monitoring and management. Stratification analysis according to sex further demonstrated this correlation.

To date, the precise mechanisms that underlying the associations of non‐O blood type and elevated Lp(a) levels with the risk of CVD are not yet fully understood. The ABO blood group system was originally thought to be specific to red blood cells, but subsequently it has been recognized that its carbohydrate structures (A, B, and H determinants) are also expressed on other cell types, including endothelial cells and platelets [30]. Intriguingly, recent studies supported that ABO blood group system affects multiple aspects of plasma coagulation proteins, including VWF biology, factor VIII, and thrombomodulin, and specific aspects of platelet function, resulting in an increased thrombotic risk in individuals with non‐O blood group [30, 42]. As is well known, blood group O results from a deletion of guanine‐258 near the protein's N‐terminus. This deletion induces a frameshift mutation. Consequently, the translated protein lacks glycosyltransferase activity. This, in turn, leads to lower circulating levels of VWF and Factor VIII compared to non‐O blood groups [12]. Moreover, the ABO blood group is responsible for 30% of the heritable variation in VWF levels [43]. Thus, plasma VWF levels are about 25% lower in blood group O compared with non‐O blood group. It's suggested that VWF in blood group O is cleaved more rapidly by ADAMTS13 and its interaction with platelets may also be reduced compared to non‐O blood type [30, 44]. In addition, previous GWAS have demonstrated genetic variants in *ABO* to be linked to the levels of various inflammatory markers, including CRP, ICAM‐1, vascular endothelial growth factor receptor 2 (VEGFR‐2), P‐selectin, E‐selectin, and so on, with an elevated inflammatory status in subjects with non‐O group [9, 42]. Similarly, as indicated above, the structural features of Lp(a) may endow it with the physiological functions of promoting atherosclerosis and thrombosis. This effect is likely derived not only from the presence of apolipoprotein B100, but also from apo(a) structure that exhibits significant homology with plasminogen, potentially altering hemostatic activity. Furthermore, Lp(a) acts as the primary lipoprotein carrier for oxidized phospholipids, which adversely impact vascular inflammation, atherosclerotic lesions, endothelial function, and thrombogenicity, ultimately leading to CVEs [45]. Notably, non‐O blood group and elevated Lp(a) may share certain underlying mechanisms that contribute to an increased risk of CVD.

Nowadays, the combined evaluation and management of multiple risk factors for stratifying and reducing cardiovascular risk has been appreciated increasingly. It was observed that the coexistence of two or more risk factors was generally correlated with an increased risk of CVD [46], and intriguingly, a synergistic rather than additive increase in risk often occurred [47]. Indeed, the combination of elevated Lp(a) levels with another risk factor, such as hypertension, dysglycemia, systemic inflammation, and family history of CAD, greatly enhanced the risk of CVEs [22, 25, 26, 28]. However, in the Utrecht Cardiovascular Cohort—Second Manifestations of ARTerial diseases (UCC‐SMART) study, based on the patterns of the clustering of four modifiable risk factors (dyslipidemia, hypertension, overweight, and smoking), the smoking–hypertension–dyslipidemia combination, instead of combining all four, was associated with the highest risk and the combination of hypertension and dyslipidemia possessed the highest population attributable fraction [46]. Peters et al. reported similar findings as well [48]. Thus, the combination of more risk factors is not always associated with higher cardiovascular risk, and the exploration of specific patterns of combination is of great significance, which has become a hot research topic in cardiovascular medicine. In view of the independent association of ABO blood group and Lp(a) with the risk of CVEs and their common pathogenic characteristics, we hypothesized that there may be a close correlation between ABO blood type and plasma Lp(a) levels in predicting the risk of MACEs. Indeed, in the present study, with two large CCS cohorts, we observed that when categorizing according to ABO blood groups and Lp(a) levels together, patients with non‐O blood type plus high Lp(a) concentrations were at the greatest risk of MACEs, followed by patients with non‐O blood group and medium levels of Lp(a), and then those with blood group O plus high Lp(a) levels. There was a significant interaction between ABO blood group and Lp(a) in predicting the risk of MACEs.

However, several limitations should be considered when interpreting our findings. First, as an observational study, a causal correlation of non‐O blood groups and elevated Lp(a) levels with the MACEs burden cannot be established. Second, it is worth noting that patients with blood group O exhibited the lowest prevalence of previous MI, a known risk factor for subsequent coronary events. Whereas, prior MI had been adjusted for in the multivariate Cox regression analysis, and this finding also reflects that individuals with blood group O had lower cardiovascular risk. Third, although major covariates were adjusted in the multivariate Cox regression models, unmeasured confounders (e.g., dietary habits, socioeconomic status, or genetic variants beyond *ABO*) may influence outcomes. Fourth, the dates of data collection of the two independent cohorts overlap slightly. However, the two cohorts of patients were recruited from two independent centers and very few patients appeared in both centers. Regarding the few patients who appeared in both cohorts, the same patient with a later admission date was excluded to avoid repetition. Fifth, as all participants in this study were of Chinese ethnicity, a subgroup analysis by race was not feasible, which may limit the external validity and generalizability of our findings. Sixth, hsCRP is included in the Cox regression analysis, but other inflammatory markers (e.g., interleukin‐6, fibrinogen, etc.) linked to ABO blood group/Lp(a) interactions are not available in our study. Finally, the mean follow‐up duration may be insufficient to capture late MACEs, which might influence our conclusions. However, the Kaplan–Meier curves showed that the difference of the MACEs risk between non‐O group‐medium or high Lp(a) and O group‐Low Lp(a) (reference) groups became more pronounced over time, hence extended follow‐up may further confirm our findings. Meanwhile, we are continuing to follow up these patients and hope to demonstrate our findings with longer‐term data. In addition, future studies with a larger sample size and longer follow‐up time may be necessary to clarify the association between AB blood group and the risk of MACEs.

In conclusion, our data demonstrated for the first time a more prominent association between Lp(a) and adverse outcomes in CCS patients with non‐O blood group compared to those with blood group O. Furthermore, the coexistence of A or B blood group and elevated Lp(a) levels was strongly associated with worse cardiovascular outcomes among patients with CCS. This is not merely an academic finding but has tangible clinical utility. By adopting this targeted, cost‐effective screening strategy focused on patients with established CAD, clinicians may be able to move beyond traditional risk calculators. This is expected to bring about a more personalized, biology‐informed approach to CVEs prevention, ultimately enabling earlier intervention for high‐risk individuals and improving their outcomes. Future RCTs are needed to validate this screening protocol and determine whether intensive intervening based on this algorithm could reduce MACEs.

## 4 Materials and Methods

### 4.1 Study Population

In this prospective cohort study, all procedures were performed in compliance with the ethical standards of the institutional research committee (Fuwai Hospital & National Center for CVDs, Beijing, China) and the 1964 Helsinki and its later amendments. This study also strictly followed the data anonymization protocols. Informed consent was obtained from all individuals enrolled in this study.

**FIGURE 3:**
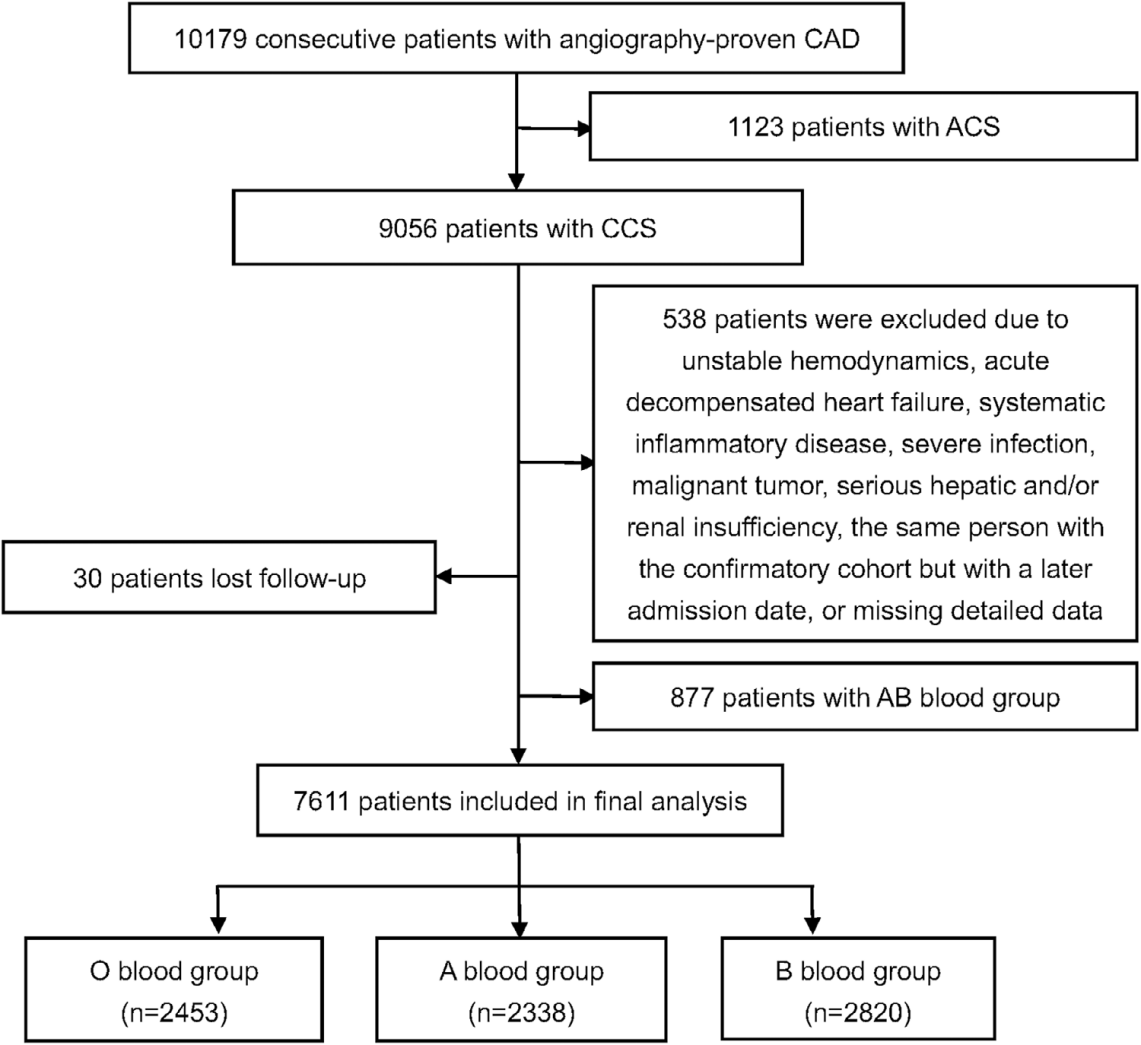
The flow chart illustrating the exploratory cohort (*n* = 7611) CAD, coronary artery disease; ACS, acute coronary syndrome; CCS, chronic coronary syndrome.

From March 2011 to July 2017, a total of 10,179 patients with CAD confirmed by angiography were consecutively recruited from the Cardiometabolic Center of Fuwai Hospital. Of these, 1123 patients were excluded due to ACS. Subsequently, 538 patients with the following conditions were excluded: unstable hemodynamics, acute decompensated heart failure, systemic inflammatory disease, severe infection, malignant tumor, serious hepatic and/or renal insufficiency, the same person as in the confirmatory cohort but with a later admission date, or missing detailed data. There were 30 individuals lost to follow‐up. In addition, referring to previous studies [5], 877 patients with blood group AB were excluded due to their relatively small sample size compared to O, A, and B blood groups to avoid drawing incorrect conclusions. Finally, 7611 patients with CCS were included in the analysis, serving as the exploratory cohort (Figure 3). Furthermore, another independent cohort of patients (*n *= 7916) with CCS, recruited from the Center for Coronary Heart Disease of Fuwai hospital between January 2017 and December 2018, was used as the confirmatory cohort to validate the findings of the exploratory cohort (Figure S5). This population is described in detail in the Supplemental Materials. All patients were categorized according to ABO blood groups (O, A, and B) and Lp(a) levels (Low: < 10 mg/dL, Medium: ≥ 10 and < 30 mg/dL, and High: ≥ 30 mg/dL) [31, 49, 50] separately, and then combinedly.

### 4.2 Clinical Assessment

Baseline information on medical histories, demographic characteristics, personal habits, and biochemical indices for all individuals were recorded. Current smoking status referred to individuals who had been regularly smoking within the past 12 months. The diagnosis of DM was based on current use of hypoglycemic agents, HbA1c ≥ 6.5%, fasting plasma glucose ≥ 7.0 mmol/L, or 2‐h plasma glucose during an oral glucose tolerance test ≥ 11.1 mmol/L. Hypertension referred to either current use of antihypertensive medications or consecutively measured SBP ≥ 140 mmHg and/or DBP ≥ 90 mmHg for three or more occasions.

### 4.3 Laboratory Analysis

Venous blood samples were obtained from all participants in the morning after more than 12‐h of fasting. As reported in our prior works [4, 19, 22], ABO blood groups (O, A, B, and AB) were detected by agglutination techniques complying with standard procedures. Lipid profiles were determined using an automatic biochemistry analyzer (Hitachi 7150, Tokyo, Japan). An immunoturbidimetry method [LASAY Lp(a) auto, SHIMA Laboratories Co., Ltd] with a validated Lp(a) protein standard was used to detect plasma Lp(a) concentrations. The upper limit of normal Lp(a) was 30 mg/dL and the intra‐ and inter‐assay coefficients of variation were < 10%. LDL‐C levels were measured using a homogeneous method (LDL‐C test kit, Kyowa Medex, Tokyo, Japan) with a coefficient of variation < 5%, total imprecision < 10%, and a detection limit of 0.026 mmol/L. High‐density lipoprotein cholesterol concentrations were analyzed by selective solubilization method (Determiner L HDL, Kyowa Medex, Tokyo, Japan) with a coefficient of variation less than 5%, total imprecision less than 10% and a detection limit of 0.026 mmol/L. Other biomarkers were determined using standard commercial kits conforming to standard methods.

### 4.4 Follow‐Up

The follow‐up was conducted at 6‐month intervals by well‐trained researchers via clinical visits and/or telephone calls. The composite of cardiovascular death, nonfatal MI, and ischemic stroke was defined as MACEs. Cardiovascular mortality referred to death primarily caused by AMI, sudden cardiac death, heart failure, stroke, and other cardiovascular causes (malignant arrythmia, coronary intervention, etc.). The diagnosis of nonfatal MI was in accordance to increased cardiac troponins plus characteristic symptoms or specific dynamic changes in ECG. Ischemic stroke was defined based on consistent neurological disorder accompanied by computed tomography and/or magnetic resonance imaging manifestations of acute cerebral infarction. The follow‐up period was calculated from the enrollment to the incident of MACEs or the date of last follow‐up. The assessment of each MACE was performed by three trained cardiologists blinded to the baseline information.

### 4.5 Statistical Analysis

Continuous variables are presented as mean ± standard deviation or as median with interquartile range, and Student's *t*‐test, analysis of variance, and nonparametric test were used to compare differences between groups. Categorical variables are expressed as numbers with percentages, and the chi‐square test was applied to compare the intergroup difference. Cumulative event‐free survival rates across ABO blood groups and Lp(a) levels were analyzed using the Kaplan–Meier method and compared with the log‐rank test. To investigate the joint associations of ABO blood group and Lp(a) levels with the risk of MACEs, patients were categorized into six groups: Group 1, O blood group and low Lp(a) level (reference group); Group 2, O blood group and medium Lp(a) level; Group 3, O blood group and high Lp(a) level; Group 4, Non‐O blood group and low Lp(a) level; Group 5, Non‐O blood group and medium Lp(a) level; and Group 6, Non‐O blood group and high Lp(a) level. Cox proportional hazards models were used to further assess the individual and combined associations of ABO blood group and Lp(a) levels with MACEs. Covariates, including sex, age, body mass index, DM, hypertension, prior MI, prior revascularization, current smoking, LDL‐C, triglyceride, hsCRP, creatinine, LVEF, statin use, ABO blood group, and Lp(a), other than the variables being analyzed, were adjusted in the models. Schoenfeld residuals analyses were used to validate the Cox proportional hazards model assumptions. Furthermore, the individual and combined associations of ABO blood types and Lp(a) with cardiovascular death, nonfatal MI, and ischemic stroke were evaluated, respectively. Stratification analyses according to O/non‐O blood groups and interaction analysis between Lp(a) and ABO blood types were conducted to examine whether the relation of Lp(a) to the risk of MACEs would be modified by ABO blood groups. C‐statistic analysis was used to examine the changes in predictive efficacy by adding ABO blood type and/or Lp(a) to the established SCORE2 risk model [51]. RCS analyses adjusted for sex and age were performed to evaluate the trend of the association between Lp(a) and MACEs stratified by ABO blood groups. In addition, subgroup analysis by sex and sensitivity analyses through including participants with AB blood group were performed to further demonstrate the associations of ABO blood group and Lp(a) levels with the risk of MACEs. For multiple comparisons in subgroup analyses, Bonferroni corrections were applied, and adjusted *p* values, were reported where applicable. A two‐tailed *p* value of < 0.05 was considered statistically significant for all analyses. In this study, SPSS 26.0 (SPSS Inc., Chicago, IL, USA), R language version 4.2.0 (Feather Spray), and STATA 15.1 (StataCorp, College Station, TX, USA) were used for statistical analyses.

## Author Contributions

H.H.L. and C.X.S. designed the study, analyzed and interpreted data, and drafted the original manuscript. S.L., Y.Z., D.Y., W.H.S., Y.L.G., C.G.Z., N.Q.W., R.X.X., Q.D., and J.Q. conducted the study and collected data. Y.H.Z. and K.F.D. supervised the study, interpreted data, and made critical revisions to the paper. J.J.L. developed the idea, designed the study, and made critical revisions to the manuscript. All authors have read and approved the final manuscript.

## Funding

This study was supported by the CAMS Innovation Fund for Medical Sciences (CIFMS) [2025‐I2M‐C&T‐B‐022; 2021‐I2M‐1‐008; 2021‐I2M‐C&T‐B‐030], the Capital Health Development Fund [201614035], and the National High level Hospital Clinical Research Funding [2023‐GSP‐RC‐09; 2023‐GSP‐QN‐8]. The funding sources had no involvement in the study's design, data collection and analysis, decision to publish, or the manuscript's preparation.

## Ethics Statement

In this study, all procedures involving human were performed according to the ethical standards of the institutional research committee (Fuwai Hospital & National Center for Cardiovascular Diseases, Beijing, China; Approval Number: 2013‐442 and 2021‐CXGC03‐1).

## Conflicts of Interest

The authors declare no conflicts of interest.

## Supporting information




**Supporting File 1**: mco270505‐sup‐0001‐SuppMat.docx

## Data Availability

The data that supports the findings of this study is available from the corresponding author upon reasonable request, and the final decision to approve the request is at the discretion of the institute (Fuwai Hospital, Beijing) that holds the data.
