# Supplementary material for: Lipoprotein(a), ABO Blood Types and Clinical Outcomes: Novel Findings and Clinical Implications in Patients With Chronic Coronary Syndrome
Source: MedComm (2020). 2025 Nov 28;6(12):e70505. doi: 10.1002/mco2.70505 (PMC12662815; doi:10.1002/mco2.70505)
Supplement: Supplementary file 1 — Supporting File 1: mco270505‐sup‐0001‐SuppMat.docx [file MCO2-6-e70505-s001.docx]

**Supplemental Materials**

**Title:** Lipoprotein(a), ABO blood types and clinical outcomes: novel findings and clinical implications in patients with chronic coronary syndrome

**Running title:** Lp(a), ABO blood type, and MACE in CCS

**Authors:** Hui-Hui Liu^1,3#^, MD; Chen-Xi Song^2#^, MD; Sha Li^1^, MD; Yan Zhang^1^, MD; Dong Yin^2^, MD; Wei-Hua Song^2^, MD; Yuan-Lin Guo^1^, MD; Cheng-Gang Zhu^1^, MD; Na-Qiong Wu^1^, MD; Rui-Xia Xu^1^, PhD; Qian Dong^1^, MS; Jie Qian^1^, MD; Yu-Hui Zhang^3*^, Ke-Fei Dou^1,2*^, MD, PhD; Jian-Jun Li^1*^, MD, PhD

^#^The authors contributed equally to this work

**Affiliation:**

^1^Cardiometabolic Center, State Key Laboratory of Cardiovascular Disease, Fuwai Hospital, National Center for Cardiovascular Diseases, National Clinical Research Center for Cardiovascular Diseases, Chinese Academy of Medical Sciences and Peking Union Medical College;

^2^ Center for Coronary Heart Disease, State Key Laboratory of Cardiovascular Disease, Fuwai Hospital, National Center for Cardiovascular Diseases, National Clinical Research Center for Cardiovascular Diseases, Chinese Academy of Medical Sciences and Peking Union Medical College;

^3^Heart Failure Center, State Key Laboratory of Cardiovascular Disease, Fuwai Hospital, National Center for Cardiovascular Diseases, National Clinical Research Center for Cardiovascular Diseases, Chinese Academy of Medical Sciences and Peking Union Medical College

^*^**Correspondence:** Yu-Hui Zhang, E-mail: [yuhuizhangjoy@163.com](mailto:yuhuizhangjoy@163.com); Ke-Fei Dou, E-mail: [drdoukefei@126.com](mailto:drdoukefei@126.com); and Jian-Jun Li, E-mail: [lijianjun938@126.com](mailto:lijianjun938@126.com). No. 167 BeiLiShi Road, XiCheng District, Beijing, 100037, China; Tel: 86+10+88396077; Fax: 86+10 +88396584

**Supplemental Methods**

As shown in Supplemental Figure 1, from January 2017 to December 2018, a total of 11208 patients with stable angina-like symptom, no acute coronary syndrome within previous 3 months or during hospitalization, and angiographically-proven coronary stenosis ≥50% in major epicardial vessels were enrolled in the Center for Coronary Heart Disease of Fuwai Hospital. Among these patients, 50 patients with missing data on blood groups and 1,485 patients with missing data on lipoprotein(a) [Lp(a)] were excluded. Other 883 patients were excluded due to the following reasons: lost to follow-up, left ventricular ejection fraction (LVEF) ≤ 35%, cardiac shock or unstable hemodynamic status, New York Heart Association (NYHA) class III-IV heart failure, end stage renal disease on dialysis or estimated glomerular filtration rate (eGFR) <30 ml/min/1.73m^2^, the same patient with the exploratory cohort but with a later admission date, or missing detailed data. Patients with AB blood group (n=874) were excluded due to its small sample size. The resulting study population included 7,916 patients with chronic coronary syndrome (CCS).

**Supplemental Table 1. Baseline characteristics of the confirmatory cohort according to ABO blood group**

| **Variable** | **Overall**  **(n=7916)** | **Blood groups**† | | | ***p* value** |
| --- | --- | --- | --- | --- | --- |
|  |  | **O (n=2514)** | **A (n=2456)** | **B (n=2946)** |  |
| Age, years | 59.8±9.6 | 60.1±9.6 | 59.6±9.6 | 59.9±9.5 | 0.216 |
| Man, n (%) | 5577 (70.5) | 1749 (69.6) | 1767 (72.0) | 2061 (69.96) | 0.141 |
| BMI, kg/m^2^ | 25.88±3.35 | 25.91±3.38 | 25.82±3.31 | 25.91±3.35 | 0.591 |
| Current smokers, n (%) | 3082 (38.9) | 993 (39.5) | 967 (39.4) | 1122 (38.1) | 0.490 |
| DM, n (%) | 2325 (29.4) | 743 (29.6) | 706 (28.8) | 876 (29.7) | 0.708 |
| Hypertension, n (%) | 4948 (62.5) | 1613 (64.2) | 1533 (62.4) | 1802 (61.2) | 0.074 |
| SBP, mmHg | 131±17 | 132±17^a^ | 130±17^a^ | 131±17 | 0.027 |
| DBP, mmHg | 78±11 | 78±11 | 77±11 | 78±11 | 0.390 |
| Family history of CAD, n (%) | 846 (10.7) | 295 (11.7) | 272 (11.1) | 279 (9.5) | 0.020 |
| Pre-MI, n (%) | 948 (12.0) | 273 (11.0) | 293 (11.9) | 382 (13.0) | 0.057 |
| Pre-RV, n (%) | 1035 (13.1) | 304 (12.1) | 333 (13.6) | 398 (13.5) | 0.209 |
| LVEF, % | 63.18±6.14 | 62.99±3.70 | 63.20±3.49 | 63.01±3.85 | 0.082 |
| Biochemical parameters | | | | | |
| TC, mmol/L | 4.11±1.08 | 4.06±1.03^a^ | 4.20±1.11^a,b^ | 4.07±1.09^b^ | 0.001 |
| HDL-C, mmol/L | 1.18±0.32 | 1.16±0.32 | 1.18±0.32 | 1.18±0.32 | 0.059 |
| LDL-C, mmol/L | 2.47±0.92 | 2.44±0.89^a^ | 2.55±0.94^a,b^ | 2.43±0.93^b^ | 0.001 |
| TG, mmol/L | 1.41 (1.04-1.95) | 1.41 (1.04-1.92) | 1.41 (1.03-1.03) | 1.41(1.04-1.97) | 0.964 |
| Lp(a), mg/dL | 15.27 (6.50-37.40) | 15.19 (6.54-37.49) | 16.36 (6.92-37.79) | 14.50 (6.11-36.64) | 0.214 |
| Lp(a) >50 mg/dL, n (%) | 1432 (18.1) | 448 (17.8) | 461 (18.8) | 523 (17.8) | 0.570 |
| ApoAI, g/L | 1.43±0.29 | 1.42±0.29^a^ | 1.43±0.29 | 1.44±0.30^a^ | 0.037 |
| ApoB, g/L | 0.76±0.23 | 0.76±0.22^a^ | 0.78±0.23^a,b^ | 0.76±0.23^b^ | 0.001 |
| FPG, mmol/L | 6.33±1.12 | 6.36±1.16 | 6.34±1.12 | 6.29±1.08 | 0.124 |
| HbA1c, % | 6.27±2.12 | 6.33±2.23 | 6.24±2.09 | 6.25±2.05 | 0.211 |
| hsCRP, mg/L | 1.26 (0.61-2.47) | 1.24 (0.58-2.37) | 1.30 (0.65-2.53) | 1.26 (0.60-2.50) | 0.285 |
| Creatinine, umol/L | 81.41±16.47 | 81.62±18.65 | 81.41±15.81 | 81.22±14.97 | 0.858 |
| Medications at discharge | | | | | |
| Antiplatelet drugs, n (%) | 7734 (97.7) | 2459 (97.8) | 2397 (97.6) | 2878 (97.7) | 0.824 |
| Statins, n (%) | 7539 (95.2) | 2395 (95.3) | 2331 (94.9) | 2813 (95.5) | 0.612 |
| β-blockers, n (%) | 6462 (78.5) | 2058(81.9) | 2005(81.6) | 2399 (81.4) | 0.920 |
| CCB, n (%) | 2735 (34.6) | 873(34.7) | 843(34.3) | 1019 (34.6) | 0.955 |

Continuous values are summarized as mean ± SD, median (interquartile range) and categorical variables as percentage. BMI, body mass index; DM, Diabetes mellitus; SBP, systolic blood pressure; DBP, diastolic blood pressure; CAD, coronary artery disease; Pre-MI, previous myocardial infarction; Pre-RV, previous revascularization; LVEF, left ventricular ejection fraction; TC, total cholesterol; HDL-C, high-density lipoprotein cholesterol; LDL-C, low-density lipoprotein cholesterol; TG, triglyceride; Lp(a), lipoprotein(a); ApoAI, apolipoprotein AI; ApoB, apolipoprotein B; FPG, fasting plasma glucose; HbA1c, glycosylated hemoglobin; hsCRP, high-sensitivity C-reactive protein; CCB, calcium channel blockers. The analysis of variance, nonparametric test, and chi square test were used to compare the differences between groups as appropriate.

†Excluded AB blood group due to its small sample size.

**Supplemental Table 2. Characteristics of the patients with or without MACEs in the exploratory cohort**

| **Variables** | **Without MACEs (n=7051)** | **With MACEs**  **(n=560)** | ***p* value** |
| --- | --- | --- | --- |
| Age, years | 57.2±10.6 | 62.1±10.2 | <0.001 |
| Male, n (%) | 5108 (72.4) | 401 (71.6) | 0.673 |
| BMI, kg/m^2^ | 25.88±3.18 | 25.61±3.21 | 0.111 |
| Current smokers, n (%) | 2998 (42.5) | 217 (38.8) | 0.144 |
| DM, n (%) | 1909 (27.1) | 201 (35.9) | <0.001 |
| Hypertension, n (%) | 4325 (61.3) | 389 (69.5) | 0.001 |
| SBP, mmHg | 126±17 | 129±18 | 0.022 |
| DBP, mmHg | 78±11 | 76±11 | 0.022 |
| Family history of CAD, n (%) | 980 (13.9) | 70 (12.5) | 0.409 |
| Pre-MI, n (%) | 2025 (28.7) | 225 (40.2) | <0.001 |
| Pre-RV, n (%) | 2003 (28.4) | 184 (32.9) | 0.054 |
| Blood groups† |  |  | 0.034 |
| O group | 2302 (32.6) | 151 (27.0) |  |
| A group | 2150 (30.5) | 188 (33.6) |  |
| B group | 2599 (36.9) | 221 (39.4) |  |
| LVEF, % | 63.62±7.86 | 60.24±10.88 | <0.001 |
| TC, mmol/L | 4.13±1.16 | 4.18±1.28 | 0.386 |
| HDL-C, mmol/L | 1.05±0.29 | 1.05±0.29 | 0.805 |
| LDL-C, mmol/L | 2.50±1.00 | 2.51±1.11 | 0.835 |
| TG, mmol/L | 1.49 (1.10-2.09) | 1.50 (1.05-2.11) | 0.820 |
| Lp(a), mg/dL | 14.85 (6.63-35.86) | 20.44 (9.15-45.56) | <0.001 |
| Lp(a) >50 mg/dL, n (%) | 1214 (16.8) | 84 (22.6) | 0.003 |
| ApoAI, g/L | 1.33±0.29 | 1.34±0.30 | 0.578 |
| ApoB, g/L | 0.91±0.30 | 0.93±0.31 | 0.214 |
| FPG, mmol/L | 5.85±1.76 | 5.99±1.97 | 0.129 |
| HbA1c, % | 6.31±1.10 | 6.62±1.26 | <0.001 |
| hsCRP, mg/L | 1.35 (0.74-2.80) | 1.74 (0.91-3.74) | <0.001 |
| Creatinine, umol/L | 77.87±18.33 | 80.84±19.33 | 0.001 |
| Medications at discharge |  |  |  |
| Antiplatelet drugs, n (%) | 6892 (97.7) | 543 (97.0) | 0.424 |
| Statins, n (%) | 6723 (95.3) | 540 (96.4) | 0.297 |
| β-blockers, n (%) | 5520 (78.3) | 453 (80.9) | 0.221 |
| CCB, n (%) | 2759 (39.1) | 216 (38.6) | 0.867 |

Continuous values are summarized as mean ± SD, median (interquartile range) and categorical variables as percentage. BMI, body mass index; DM, Diabetes mellitus; SBP, systolic blood pressure; DBP, diastolic blood pressure; CAD, coronary artery disease; Pre-MI, previous myocardial infarction; Pre-RV, previous revascularization; LVEF, left ventricular ejection fraction; TC, total cholesterol; HDL-C, high-density lipoprotein cholesterol; LDL-C, low-density lipoprotein cholesterol; TG, triglyceride; Lp(a), lipoprotein(a); ApoAI, apolipoprotein AI; ApoB, apolipoprotein B; FPG, fasting plasma glucose; HbA1c, [glycosylated](javascript:void(0);) [hemoglobin](javascript:void(0);); hsCRP, high-sensitivity C-reactive protein; CCB, calcium channel blockers. The student’s t-test, nonparametric test, and chi square test were used to compare the differences between groups as appropriate.

†Excluded AB blood group due to its small sample size.

**Supplemental Table 3. Univariate and multivariate Cox regression analyses for MACEs among patients in the exploratory cohort**

| Variables | Univariate analysis | |  | Multivariate analysis | |
| --- | --- | --- | --- | --- | --- |
|  | **HR (95% CI)** | ***p* value** |  | **HR (95% CI)** | ***p* value** |
| ABO blood group† |  |  |  |  |  |
| O group | 1.00 (reference) | - |  | 1.00 (reference) | - |
| A group | 1.36 (1.04-1.78) | 0.026 |  | 1.36 (1.02-1.81) | 0.044 |
| B group | 1.33 (1.02-1.73) | 0.035 |  | 1.37 (1.04-1.81) | 0.026 |
| Lp(a) |  |  |  |  |  |
| Low Lp(a) | 1.00 (reference) | - |  | 1.00 (reference) | - |
| Medium Lp(a) | 1.55 (1.20-2.00) | 0.001 |  | 1.51 (1.15-1.98) | 0.004 |
| High Lp(a) | 1.74 (1.34-2.28) | <0.001 |  | 1.71 (1.31-2.25) | <0.001 |
| Age | 1.05 (1.03-1.06) | <0.001 |  | 1.04 (1.03-1.06) | <0.001 |
| Sex | 0.93 (0.74-1.17) | 0.538 |  | 1.12 (0.85-1.49) | 0.431 |
| BMI | 0.97 (0.94-1.01) | 0.103 |  | 0.98 (0.94-1.01) | 0.976 |
| Hypertension | 1.34 (1.07-1.68) | 0.010 |  | 1.33 (1.04-1.71) | 0.025 |
| Diabetes mellitus | 1.49 (1.19-1.85) | <0.001 |  | 1.41 (1.12-1.78) | 0.003 |
| Current smoking | 0.84 (0.68-1.05) | 0.123 |  | 0.86 (0.66-1.11) | 0.237 |
| Pre-MI | 1.67 (1.35-2.08) | <0.001 |  | 1.35 (1.04-1.75) | 0.025 |
| Pre-RV | 1.20 (0.96-1.51) | 0.104 |  | 1.03 (0.81-1.31) | 0.832 |
| LDL-C | 1.05 (0.95-1.17) | 0.315 |  | 1.07 (0.96-1.20) | 0.241 |
| TG | 1.01 (0.93-1.10) | 0.773 |  | 1.09 (1.02-1.17) | 0.012 |
| hsCRP | 1.01 (1.00-1.02) | 0.016 |  | 1.01 (1.00-1.02) | 0.161 |
| Creatinine | 1.01 (1.00-1.01) | <0.001 |  | 1.00 (1.00-1.01) | 0.401 |
| LVEF | 0.96 (0.95-0.97) | <0.001 |  | 0.96 (0.95-0.97) | <0.001 |
| Statin use | 1.15 (0.63-2.09) | 0.659 |  | 1.14 (0.61-2.16) | 0.681 |

Events = 560, Total participants = 7611. MACEs, major adverse cardiovascular events; CCS, chronic coronary syndrome; HR, hazard ratio; CI, confidence interval; Lp(a), lipoprotein(a); BMI, body mass index; Pre-MI, previous myocardial infarction; Pre-RV, previous revascularization; LDL-C, low-density lipoprotein cholesterol; TG, triglyceride; hsCRP, high-sensitivity C-reactive protein; LVEF, left ventricular ejection fraction. The significance was tested by univariate and multivariate Cox regression analyses. †Excluded AB blood group due to its small sample size.

**Supplemental Table 4. Schoenfeld residuals analyses for validating the Cox proportional hazards model assumption in the exploratory cohort**

| **Variables** | **χ²** | ***p* value** |
| --- | --- | --- |
| ABO blood group† | 2.99 | 0.224 |
| Lp(a) group | 4.97 | 0.084 |
| Sex | 1.06 | 0.303 |
| Age | 0.46 | 0.499 |
| BMI | 0.44 | 0.508 |
| Hypertension | 3.09 | 0.079 |
| DM | 1.91 | 0.167 |
| Current smoking | 0.16 | 0.691 |
| Pre-MI | 0.70 | 0.403 |
| Pre-RV | 0.01 | 0.967 |
| LDL-C | 2.07 | 0.150 |
| TG | 0.07 | 0.794 |
| hsCRP | 2.24 | 0.135 |
| Creatinine | 0.97 | 0.324 |
| LVEF | 0.10 | 0.747 |
| Statin use | 0.02 | 0.897 |
| Global | 24.96 | 0.126 |

Events = 560, Total participants = 7611. BMI, body mass index; DM, Diabetes mellitus; Pre-MI, previous myocardial infarction; Pre-RV, previous revascularization; LVEF, left ventricular ejection fraction; LDL-C, low-density lipoprotein cholesterol; TG, triglyceride; Lp(a), lipoprotein(a); hsCRP, high-sensitivity C-reactive protein.

†Excluded AB blood group due to its small sample size.

**Supplemental Table 5.** **Multivariate Cox regression analyses of ABO blood group and Lp(a) for predicting cardiovascular death, non-fatal MI, or ischemic stroke in the exploratory cohort**

| Category | Adjusted HR (95% CI) | | |
| --- | --- | --- | --- |
|  | **Cardiovascular death (n=221)** | **Non-fatal MI (n=117)** | **Ischemic stroke**  **(n=222)** |
| ABO blood group† |  |  |  |
| O group | 1.00 (reference) | 1.00 (reference) | 1.00 (reference) |
| A group | 1.43 (0.92-2.23) | 0.82 (0.43-1.55) | 1.64 (1.02-2.66)^*^ |
| B group | 1.29 (0.83-1.97) | 1.24 (0.71-2.15) | 1.57 (0.99-2.51) |
| Lp(a) |  |  |  |
| Low Lp(a) | 1.00 (reference) | 1.00 (reference) | 1.00 (reference) |
| Medium Lp(a) | 1.62 (1.05-2.51)^*^ | 1.30 (0.71-2.37) | 1.40 (0.89-2.20) |
| High Lp(a) | 1.68 (1.08-2.62)^*^ | 1.63 (0.91-2.95) | 1.65 (1.05-2.60)^*^ |
| Per 1-SD increase of LgLp(a) | 1.23 (1.03-1.47)^*^ | 1.17 (0.91-1.50) | 1.18 (0.98-1.42) |
| ABO blood group† and Lp(a) |  |  |  |
| O group-Low Lp(a) | 1.00 (reference) | 1.00 (reference) | 1.00 (reference) |
| O group-Medium Lp(a) | 1.32 (0.52-3.35) | 0.63 (0.21-1.84) | 1.66 (0.63-4.38) |
| O group-High Lp(a) | 2.22 (0.98-5.03) | 0.86 (0.33-2.28) | 1.81 (0.70-4.70) |
| Non-O group-Low Lp(a) | 1.45 (0.68-3.11) | 0.51 (0.21-1.23) | 1.81 (0.79-4.17) |
| Non-O group-Medium Lp(a) | 2.46 (1.19-5.09)^*^ | 0.99 (0.46-2.17) | 2.41 (1.06-5.46)^*^ |
| Non-O group-High Lp(a) | 2.19 (1.04-4.63)^*^ | 1.22 (0.56-2.64) | 2.92 (1.29-6.62)^*^ |

Total participants = 7611. Adjusted model adjusted for sex, age, body mass index, hypertension, diabetes mellitus, current smoking, prior myocardial infarction, prior revascularization, low-density lipoprotein cholesterol, triglyceride, high-sensitivity C-reactive protein, creatinine, left ventricular ejection fraction, statin use, ABO blood group, and Lp(a), other than the variables being analyzed. Lp(a), lipoprotein(a); LgLp(a), log-transformed Lp(a); MACEs, major adverse cardiovascular events; HR, hazard ratio; CI, confidence interval. The significance was tested by multivariate Cox regression analyses. †Excluded AB blood group due to its small sample size; ^*^*p*<0.05.

**Supplemental Table 6. Subgroup analyses of the association of Lp(a) with cardiovascular death, non-fatal MI, and ischemic stroke respectively according to ABO blood group in the exploratory cohort**

| ABO blood group† | Lp(a) levels | Adjusted Model HR (95% CI) | | |
| --- | --- | --- | --- | --- |
|  |  | **Cardiovascular death (n=221)** | **Non-fatal MI (n=117)** | **Ischemic stroke (n=222)** |
| O group | Low Lp(a) | 1.00 (reference) | 1.00 (reference) | 1.00 (reference) |
|  | Medium Lp(a) | 1.34 (0.69-2.61) | 0.67 (0.31-1.45) | 1.58 (0.80-3.15) |
|  | High Lp(a) | 2.00 (1.10-3.62)^*^ | 0.87 (0.43-1.76) | 1.80 (0.91-3.55) |
|  | Per 1-SD increase of LgLp(a) | 1.36 (1.06-1.76)^*^ | 0.91 (0.67-1.22) | 1.24 (0.94-1.64) |
| Non-O group† | Low Lp(a) | 1.00 (reference) | 1.00 (reference) | 1.00 (reference) |
|  | Medium Lp(a) | 1.59 (1.12-2.27)^*^ | 2.18 (1.23-3.86)^*^ | 1.27 (0.88-1.84) |
|  | High Lp(a) | 1.46 (1.01-2.12)^*^ | 2.72 (1.54-4.82)^*^ | 1.54 (1.07-2.23)^*^ |
|  | Per 1-SD increase of LgLp(a) | 1.14 (0.98-1.33) | 1.39 (1.11-1.74)^*^ | 1.13 (0.97-1.32) |

Total participants = 7611. Adjusted model adjusted for sex, age, body mass index, hypertension, diabetes mellitus, current smoking, prior myocardial infarction, prior revascularization, low-density lipoprotein cholesterol, triglyceride, high-sensitivity C-reactive protein, creatinine, left ventricular ejection fraction, and statin use. Lp(a), lipoprotein(a); LgLp(a), log-transformed Lp(a); MI, myocardial infarction; HR, hazard ratio; CI, confidence interval. The significance was tested by multivariate Cox regression analyses.

†Excluded AB blood group due to its small sample size; ^*^*p*<0.05.

**Supplemental Table 7. Characteristics of the patients with or without MACEs in the confirmatory cohort**

| **Variables** | **Without MACEs**  **(n=7662)** | **With MACEs**  **(n=254)** | ***p* value** |
| --- | --- | --- | --- |
| Age, years | 59.7±9.5 | 63.0±11.4 | <0.001 |
| Man, n (%) | 5396 (70.4) | 181 (71.3) | 0.774 |
| BMI, kg/m^2^ | 25.87±3.19 | 25.77±4.05 | 0.677 |
| Current smokers, n (%) | 2985 (39.0) | 97 (38.2) | 0.805 |
| DM, n (%) | 2241 (29.2) | 84 (33.1) | 0.188 |
| Hypertension, n (%) | 4782 (62.4) | 166 (65.4) | 0.341 |
| SBP, mmHg | 131±17 | 132±19 | 0.331 |
| DBP, mmHg | 77±11 | 77±11 | 0.516 |
| Family history of CAD, n (%) | 823 (10.7) | 23 (9.1) | 0.392 |
| Pre-MI, n (%) | 903 (11.8) | 45 (17.7) | 0.004 |
| Pre-RV, n (%) | 986 (12.9) | 49 (19.3) | 0.003 |
| Blood groups† |  |  | 0.026 |
| O group | 2452 (32.0) | 62 (24.4) |  |
| A group | 2363 (30.8) | 93 (36.6) |  |
| B group | 2847 (37.2) | 99 (39.0) |  |
| LVEF, % | 63.09±3.64 | 62.28±5.10 | 0.012 |
| TC, mmol/L | 4.11±1.07 | 4.03±1.23 | 0.297 |
| HDL-C, mmol/L | 1.18±0.32 | 1.15±0.33 | 0.330 |
| LDL-C, mmol/L | 2.47±0.92 | 2.45±1.01 | 0.790 |
| TG, mmol/L | 1.41 (1.04-1.95) | 1.40 (1.00-1.71) | 0.015 |
| Lp(a), mg/dL | 15.10 (6.47-37.15) | 21.10 (9.01-46.73) | <0.001 |
| Lp(a) >50 mg/dL, n (%) | 1372 (17.9) | 60 (23.6) | 0.020 |
| ApoAI, g/L | 1.44±0.29 | 1.38±0.30 | 0.008 |
| ApoB, g/L | 0.76±0.23 | 0.76±0.25 | 0.668 |
| FPG, mmol/L | 6.32±1.11 | 6.58±1.33 | 0.002 |
| HbA1c, % | 6.26±2.11 | 6.52±2.49 | 0.111 |
| hsCRP, mg/L | 1.27 (0.60-2.47) | 1.19 (0.81-2.45) | 0.142 |
| Creatinine, umol/L | 81.34±16.40 | 83.47±18.33 | 0.043 |
| Medications at discharge |  |  |  |
| Antiplatelet drugs, n (%) | 7495 (97.8) | 239 (94.1) | <0.001 |
| Statins, n (%) | 7304 (95.3) | 235 (92.5) | 0.039 |
| β-blockers, n (%) | 6250 (81.6) | 212 (83.5) | 0.443 |
| CCB, n (%) | 2628 (34.3) | 107 (42.1) | 0.010 |

Total participants = 7916. Continuous values are summarized as mean ± SD, median (interquartile range) and categorical variables as percentage. BMI, body mass index; DM, Diabetes mellitus; SBP, systolic blood pressure; DBP, diastolic blood pressure; CAD, coronary artery disease; Pre-MI, previous myocardial infarction; Pre-RV, previous revascularization; LVEF, left ventricular ejection fraction; TC, total cholesterol; HDL-C, high-density lipoprotein cholesterol; LDL-C, low-density lipoprotein cholesterol; TG, triglyceride; Lp(a), lipoprotein(a); ApoAI, apolipoprotein AI; ApoB, apolipoprotein B; FPG, fasting plasma glucose; HbA1c, [glycosylated](javascript:void(0);) [hemoglobin](javascript:void(0);); hsCRP, high-sensitivity C-reactive protein; CCB, calcium channel blockers. The student’s t-test, nonparametric test, and chi square test were used to compare the differences between groups as appropriate.

†Excluded AB blood group due to its small sample size; ^*^*p*<0.05.

**Supplemental Table 8. Cox regression analyses of ABO blood group and Lp(a) levels for predicting MACEs in the confirmatory cohort**

| **Category (Events/Total subjects)** | **Crude Model**  **HR (95% CI)** | **Adjusted Model**  **HR (95% CI)** |
| --- | --- | --- |
| ABO blood group† |  |  |
| O group (62/2514) | 1.00 (reference) | 1.00 (reference) |
| A group (93/2456) | 1.54 (1.12-2.13)^*^ | 1.54 (1.12-2.13)^*^ |
| B group (99/2946) | 1.37 (1.00-1.88) | 1.37 (1.00-1.89) |
| Lp(a) |  |  |
| Low Lp(a) (69/2990) | 1.00 (reference) | 1.00 (reference) |
| Medium Lp(a) (87/2478) | 1.55 (1.13-2.13)^*^ | 1.44 (1.05-1.98)^*^ |
| High Lp(a) (98/2448) | 1.82 (1.34-2.48)^**^ | 1.65 (1.21-2.26)^*^ |
| Per 1-SD increase of LgLp(a) | 1.28 (1.12-1.45)^**^ | 1.21(1.06-1.38)^*^ |
| ABO blood group† and Lp(a) |  |  |
| O group-Low Lp(a) (18/949) | 1.00 (reference) | 1.00 (reference) |
| O group-Medium Lp(a) (19/768) | 1.33 (0.70-2.54) | 1.24 (0.65-2.36) |
| O group-High Lp(a) (25/797) | 1.74 (0.95-3.19) | 1.67 (0.91-3.07) |
| Non-O group-Low Lp(a) (51/2041) | 1.33 (0.78-2.28) | 1.38 (0.81-2.36) |
| Non-O group-Medium Lp(a) (68/1710) | 2.16 (1.28-3.63)^*^ | 2.07 (1.23-3.49)^*^ |
| Non-O group-High Lp(a) (73/1651) | 2.47 (1.48-4.15)^**^ | 2.22 (1.32-3.73)^*^ |

Total participants = 7916. Adjusted model adjusted for age, sex, body mass index, hypertension, diabetes mellitus, current smoking, prior myocardial infarction, prior revascularization, low-density lipoprotein cholesterol, triglyceride, high-sensitivity C-reactive protein, creatinine, left ventricular ejection fraction, statin use, ABO blood group, and Lp(a), other than the variables being analyzed. Lp(a), lipoprotein(a); LgLp(a), log-transformed Lp(a); MACEs, major adverse cardiovascular events; HR, hazard ratio; CI, confidence interval. The significance was tested by univariate and multivariate Cox regression analyses.

†Excluded AB blood group due to its small sample size; ^*^*p*<0.05; ^**^*p*<0.001.

**Supplemental Table 9. Subgroup analyses of the association between Lp(a) and MACEs according to ABO blood group in the confirmatory cohort**

| **ABO blood group**† **(Events/Total subjects)** | **Lp(a) categories (Events/Total subjects)** | **Crude Model**  **HR (95% CI)** | **Adjusted Model**  **HR (95% CI)** |
| --- | --- | --- | --- |
| O group (62/2514) | Low Lp(a) (18/949) | 1.00 (reference) | 1.00 (reference) |
|  | Medium Lp(a) (19/768) | 1.33 (0.70-2.54) | 1.18 (0.62-2.27) |
|  | High Lp(a) (25/797) | 1.73 (0.94-3.17) | 1.55 (0.84-2.87) |
|  | Per 1-SD increase of LgLp(a) | 1.18 (0.91-1.52) | 1.11 (0.86-1.44) |
| Non-O group† (192/5402) | Low Lp(a) (51/2041) | 1.00 (reference) | 1.00 (reference) |
|  | Medium Lp(a) (68/1710) | 1.62 (1.13-2.34)^*^ | 1.50 (1.04, 2.17)^*^ |
|  | High Lp(a) (73/1651) | 1.87 (1.30-2.67)^**^ | 1.62 (1.12, 2.33)^*^ |
|  | Per 1-SD increase of LgLp(a) | 1.31 (1.13-1.52)^**^ | 1.23 (1.05-1.43)^*^ |

Total participants = 7916. Adjusted model adjusted for age, sex, body mass index, hypertension, diabetes mellitus, current smoking, prior myocardial infarction, prior revascularization, low-density lipoprotein cholesterol, triglyceride, high-sensitivity C-reactive protein, creatinine, left ventricular ejection fraction, and statin use. Lp(a), lipoprotein(a); MACEs, major adverse cardiovascular events; HR, hazard ratio; CI, confidence interval. The significance was tested by univariate and multivariate Cox regression analyses.

†Excluded AB blood group due to its small sample size; ^*^*p*<0.05; ^**^*p*<0.001.

**Supplemental Table 10. Multivariate Cox regression analyses of ABO blood group and Lp(a) for predicting cardiovascular death, non-fatal MI, or ischemic stroke in the confirmatory cohort**

| Category | Adjusted HR (95% CI) | | |
| --- | --- | --- | --- |
|  | **Cardiovascular death (n=105)** | **Non-fatal MI (n=94)** | **Ischemic stroke**  **(n=55)** |
| ABO blood group† |  |  |  |
| O group | 1.00 (reference) | 1.00 (reference) | 1.00 (reference) |
| A group | 2.74 (1.54-4.90)^**^ | 1.28 (0.80-2.05) | 1.00 (0.53-1.89) |
| B group | 2.65 (1.50-4.66)^**^ | 0.85 (0.51-1.39) | 1.13 (0.62-2.03) |
| Lp(a) |  |  |  |
| Low Lp(a) | 1.00 (reference) | 1.00 (reference) | 1.00 (reference) |
| Medium Lp(a) | 1.07 (0.66-1.74) | 1.55 (0.94-2.54) | 2.46 (1.19-5.08)^*^ |
| High Lp(a) | 1.33 (0.83-2.13) | 1.54 (0.93-2.53) | 3.23 (1.60-6.52)^*^ |
| Per 1-SD increase of LgLp(a) | 1.10 (0.90-1.35) | 1.21 (0.98-1.49) | 1.51 (1.15-1.99)^*^ |
| ABO blood group† and Lp(a) |  |  |  |
| O group-Low Lp(a) | 1.00 (reference) | 1.00 (reference) | 1.00 (reference) |
| O group-Medium Lp(a) | 0.91 (0.25-3.41) | 1.49 (0.62-3.61) | 1.78 (0.50-6.34) |
| O group-High Lp(a) | 1.73 (0.55-5.46) | 1.44 (0.59-3.49) | 2.85 (0.89-9.19) |
| Non-O group-Low Lp(a) | 2.90 (1.12-7.50)^*^ | 0.99 (0.45-2.18) | 0.83 (0.24-2.84) |
| Non-O group-Medium Lp(a) | 3.16 (1.22-8.19)^*^ | 1.55 (0.72-3.33) | 2.37 (0.79-7.08) |
| Non-O group-High Lp(a) | 3.65 (1.42-9.42)^*^ | 1.56 (0.72-3.36) | 2.86 (0.97-8.44) |

Adjusted model adjusted for age, sex, body mass index, hypertension, diabetes mellitus, current smoking, prior myocardial infarction, prior revascularization, low-density lipoprotein cholesterol, triglyceride, high-sensitivity C-reactive protein, creatinine, left ventricular ejection fraction, statin use, ABO blood group, and Lp(a), other than the variables being analyzed. Lp(a), lipoprotein(a); LgLp(a), log-transformed Lp(a); MACEs, major adverse cardiovascular events; HR, hazard ratio; CI, confidence interval. The significance was tested by multivariate Cox regression analyses. †Excluded AB blood group due to its small sample size; ^*^*p*<0.05, ^**^*p*<0.001.

**Supplemental Table 11. Subgroup analyses of the association of Lp(a) with cardiovascular death, non-fatal MI, and ischemic stroke respectively according to ABO blood group in the confirmatory cohort**

| ABO blood group† | Lp(a) levels | Adjusted Model HR (95% CI) | | |
| --- | --- | --- | --- | --- |
|  |  | **Cardiovascular death (n=105)** | **Non-fatal MI (n=94)** | **Ischemic stroke**  **(n=55)** |
| O group | Low Lp(a) | 1.00 (reference) | 1.00 (reference) | 1.00 (reference) |
|  | Medium Lp(a) | 0.92 (0.24-3.50) | 1.46 (0.60-3.59) | 1.56 (0.43-5.57) |
|  | High Lp(a) | 1.74 (0.54-5.64) | 1.36 (0.55-3.36) | 2.57 (0.80-8.27) |
|  | Per 1-SD increase of LgLp(a) | 1.23 (0.73-2.08) | 1.00 (0.70-1.44) | 1.34 (0.82-2.17) |
| Non-O group† | Low Lp(a) | 1.00 (reference) | 1.00 (reference) | 1.00 (reference) |
|  | Medium Lp(a) | 1.07 (0.64-1.82) | 1.55 (0.85-2.84) | 3.13 (1.29-7.62)^*^ |
|  | High Lp(a) | 1.24 (0.74-2.07) | 1.58 (0.86-2.89) | 3.55 (1.47-8.53)^*^ |
|  | Per 1-SD increase of LgLp(a) | 1.06 (0.85-1.33) | 1.30 (1.01-1.68)^*^ | 1.55 (1.12-2.15)^*^ |

Total participants = 7916. Adjusted model adjusted for age, sex, body mass index, hypertension, diabetes mellitus, current smoking, prior myocardial infarction, prior revascularization, low-density lipoprotein cholesterol, triglyceride, high-sensitivity C-reactive protein, creatinine, left ventricular ejection fraction, and statin use. Lp(a), lipoprotein(a); LgLp(a), log-transformed Lp(a); MI, myocardial infarction; HR, hazard ratio; CI, confidence interval. The significance was tested by multivariate Cox regression analyses.

†Excluded AB blood group due to its small sample size; ^*^*p*<0.05.

**Supplemental Table 12.** C-statistic of ABO blood group and Lp(a) for predicting MACEs in two cohorts

| Cohort (Events/N) | Models | C-statistic  (95% CI) | ΔC-statistic  (95% CI) | *p* value |
| --- | --- | --- | --- | --- |
| Exploratory cohort (560/7611) | SCORE2 risk model | 0.636 (0.603-0.668) | – |  |
|  | SCORE2 risk model + ABO blood group† | 0.646 (0.613-0.679) | 0.010 (-0.015-0.029) | 0.365 |
|  | SCORE2 risk model + Categorical Lp(a) | 0.657 (0.622-0.691) | 0.021 (-0.002-0.047) | 0.094 |
|  | SCORE2 risk model + ABO blood group† and Lp(a) | 0.668 (0.633-0.702) | 0.032 (0.004-0.057) | 0.020 |
| Confirmatory cohort (254/7916) | SCORE2 risk model | 0.613 (0.568-0.658) | – |  |
|  | SCORE2 risk model + ABO blood group† | 0.620 (0.575-0.665) | 0.007 (-0.004-0.019) | 0.207 |
|  | SCORE2 risk model + Categorical Lp(a) | 0.630 (0.589-0.672) | 0.019 (-0.002-0.037) | 0.053 |
|  | SCORE2 risk model + ABO blood group† and Lp(a) | 0.635 (0.592-0.677) | 0.023 (-0.002-0.040) | 0.030 |

SCORE2 model included age, sex, current smoking, diabetes, systolic blood pressure, total cholesterol, and high-density lipoprotein cholesterol. CI, confidence interval; Lp(a), lipoprotein(a).

**Supplemental Table 13. Sex-based subgroup analyses of ABO blood group and Lp(a) levels for predicting MACEs in the exploratory cohort**

| **Category (Events/Total subjects)** | **Adjusted Model** **HR (95% CI)** | |
| --- | --- | --- |
|  | **Men** | **Women** |
| ABO blood group† |  |  |
| O group | 1.00 (reference) | 1.00 (reference) |
| A group | 1.31 (0.94-1.84) | 1.43 (0.93-2.22) |
| B group | 1.36 (0.98-1.89) | 1.33 (0.87-2.03) |
| Lp(a) |  |  |
| Low Lp(a) | 1.00 (reference) | 1.00 (reference) |
| Medium Lp(a) | 1.66 (1.19-2.33)^*^ | 1.25 (0.81-1.93) |
| High Lp(a) | 2.03 (1.46-2.83)^**^ | 1.40 (0.90-2.16) |
| Per 1-SD increase of LgLp(a) | 1.31 (1.14-1.50)^**^ | 1.12 (0.94-1.33) |
| ABO blood group† and Lp(a) |  |  |
| O group-Low Lp(a) | 1.00 (reference) | 1.00 (reference) |
| O group-Medium Lp(a) | 1.32 (0.70-2.50) | 1.30 (0.48-3.52) |
| O group-High Lp(a) | 1.59 (0.86-2.92) | 2.63 (1.10-6.28)^*^ |
| Non-O group-Low Lp(a) | 1.05 (0.61-1.82) | 2.06 (0.90-4.70) |
| Non-O group-Medium Lp(a) | 1.92 (1.14-3.21)^*^ | 2.54 (1.13-5.69)^*^ |
| Non-O group-High Lp(a) | 2.36 (1.41-3.94)^*^ | 2.25 (0.99-5.14) |

Events = 560, Total participants = 7611. Adjusted model adjusted for age, body mass index, hypertension, diabetes mellitus, current smoking, prior myocardial infarction, prior revascularization, low-density lipoprotein cholesterol, triglyceride, high-sensitivity C-reactive protein, creatinine, left ventricular ejection fraction, statin use, ABO blood groups, and Lp(a), other than the variables being analyzed. Lp(a), lipoprotein(a); LgLp(a), log-transformed Lp(a); MACEs, major adverse cardiovascular events; HR, hazard ratio; CI, confidence interval. The significance was tested by multivariate Cox regression analyses.

†Excluded AB blood group due to its small sample size; ^*^*p*<0.05; ^**^*p*<0.001.

**Supplemental Table 14. Sex-based subgroup analyses of ABO blood group and Lp(a) levels for predicting MACEs in the confirmatory cohort**

| **Category** | **Adjusted Model** **HR (95% CI)** | |
| --- | --- | --- |
|  | **Men** | **Women** |
| ABO blood group† |  |  |
| O group | 1.00 (reference) | 1.00 (reference) |
| A group | 1.43 (0.97-2.11) | 1.93 (1.07-3.47)^*^ |
| B group | 1.43 (0.98-2.08) | 1.23 (0.67-2.27) |
| Lp(a) |  |  |
| Low Lp(a) | 1.00 (reference) | 1.00 (reference) |
| Medium Lp(a) | 1.69 (1.16-2.46)^*^ | 0.91 (0.49-1.69) |
| High Lp(a) | 1.69 (1.16-2.47)^*^ | 1.47 (0.83-2.57) |
| Per 1-SD increase of LgLp(a) | 1.23 (1.05-1.44)^*^ | 1.15 (0.90-1.48) |
| ABO blood group† and Lp(a) |  |  |
| O group-Low Lp(a) | 1.00 (reference) | 1.00 (reference) |
| O group-Medium Lp(a) | 1.45 (0.67-3.13) | 0.84 (0.26-2.79) |
| O group-High Lp(a) | 2.00 (0.96-4.16) | 1.14 (0.38-3.44) |
| Non-O group-Low Lp(a) | 1.45 (0.75-2.78) | 1.28 (0.50-3.31) |
| Non-O group-Medium Lp(a) | 2.56 (1.36-4.80)^*^ | 1.25 (0.48-3.24) |
| Non-O group-High Lp(a) | 2.31 (1.22-4.37)^*^ | 2.04 (0.83-5.03) |

Total participants = 7916. Adjusted model adjusted for age, body mass index, hypertension, diabetes mellitus, current smoking, prior myocardial infarction, prior revascularization, low-density lipoprotein cholesterol, triglyceride, high-sensitivity C-reactive protein, creatinine, left ventricular ejection fraction, statin use, ABO blood groups, and Lp(a), other than the variables being analyzed. Lp(a), lipoprotein(a); LgLp(a), log-transformed Lp(a); MACEs, major adverse cardiovascular events; HR, hazard ratio; CI, confidence interval. The significance was tested by multivariate Cox regression analyses.

†Excluded AB blood group due to its small sample size; ^*^*p*<0.05.

**Supplemental Table 15. Baseline characteristics of the patients with AB blood group in the two cohorts**

| **Variables** | **Exploratory cohort**  **(n=877)** | **Confirmatory cohort**  **(n=874)** |
| --- | --- | --- |
| Age, years | 57.5±10.8 | 59.9±9.5 |
| Man, n (%) | 625 (71.3) | 615 (70.4) |
| BMI, kg/m^2^ | 25.89±3.24 | 25.74±3.18 |
| Current smokers, n (%) | 383 (43.7) | 344 (39.4) |
| DM, n (%) | 257 (29.3) | 255 (29.2) |
| Hypertension, n (%) | 546 (62.3) | 532 (60.9) |
| SBP, mmHg | 126±18 | 132±18 |
| DBP, mmHg | 78±11 | 77±10 |
| Family history of CAD, n (%) | 140 (16.0) | 90 (10.3) |
| Pre-MI, n (%) | 274 (31.3) | 110 (12.6) |
| Pre-RV, n (%) | 243 (27.7) | 115 (13.2) |
| LVEF, % | 63.21±8.28 | 62.96±4.01 |
| TC, mmol/L | 4.23±1.18 | 4.12±1.07 |
| HDL-C, mmol/L | 1.06±0.29 | 1.17±0.31 |
| LDL-C, mmol/L | 2.59±1.04 | 2.47±0.91 |
| TG, mmol/L | 1.54 (1.13-2.11) | 1.44 (1.06-2.02) |
| Lp(a), mg/dL | 15.45 (6.93-35.99) | 15.50 (6.84-41.85) |
| Lp(a) >50 mg/dL, n (%) |  | 187 (21.4) |
| ApoAI, g/L | 1.35±0.29 | 1.43±0.28 |
| ApoB, g/L | 0.94±0.30 | 0.77±0.22 |
| FPG, mmol/L | 5.87±1.89 | 6.29±1.05 |
| HbA1c, % | 6.36±1.11 | 6.21±2.00 |
| hsCRP, mg/L | 1.47 (0.78-3.11) | 1.27 (0.62-2.56) |
| Creatinine, umol/L | 77.94±17.69 | 81.75±15.84 |
| Medications at discharge |  |  |
| Antiplatelet drugs, n (%) | 846 (97.1) | 849 (97.1) |
| Statins, n (%) | 822 (93.7) | 830 (95.0) |
| β-blockers, n (%) | 675 (77.0) | 731 (83.6) |
| CCB, n (%) | 351 (40.0) | 316 (36.2) |

Continuous values are summarized as mean ± SD, median (interquartile range) and categorical variables as percentage. BMI, body mass index; DM, Diabetes mellitus; SBP, systolic blood pressure; DBP, diastolic blood pressure; CAD, coronary artery disease; Pre-MI, previous myocardial infarction; Pre-RV, previous revascularization; LVEF, left ventricular ejection fraction; TC, total cholesterol; HDL-C, high-density lipoprotein cholesterol; LDL-C, low-density lipoprotein cholesterol; TG, triglyceride; Lp(a), lipoprotein(a); ApoAI, apolipoprotein AI; ApoB, apolipoprotein B; FPG, fasting plasma glucose; HbA1c, [glycosylated](javascript:void(0);) [hemoglobin](javascript:void(0);); hsCRP, high-sensitivity C-reactive protein; CCB, calcium channel blockers. The student’s t-test, nonparametric test, and chi square test were used to compare the differences between groups as appropriate.

†Excluded AB blood group due to its small sample size; ^*^*p*<0.05.

**Supplemental Table 16. Sensitivity analysis of the association between Lp(a) and MACEs in non-O blood group of the exploratory cohort by including AB group**

| **Lp(a) categories (Events/Total subjects)** | **Crude Model**  **HR (95% CI)** | **Adjusted Model**  **HR (95% CI)** |
| --- | --- | --- |
| Low Lp(a) (117/2241) | 1.00 (reference) | 1.00 (reference) |
| Medium Lp(a) (176/2033) | 1.60 (1.20-2.15)^*^ | 1.60 (1.17-2.18)^*^ |
| High Lp(a) (166/1505) | 1.72 (1.27-2.31)^**^ | 1.78 (1.30-2.44)^**^ |
| Per 1-SD increase of LgLp(a) | 1.21 (1.08-1.36)^*^ | 1.22 (1.08-1.39)^*^ |

Total non-O participants = 6035. Adjusted model adjusted for age, sex, body mass index, hypertension, diabetes mellitus, current smoking, prior myocardial infarction, prior revascularization, low-density lipoprotein cholesterol, triglyceride, high-sensitivity C-reactive protein, creatinine, left ventricular ejection fraction, and statin use. Lp(a), lipoprotein(a); LgLp(a), log-transformed Lp(a); MACEs, major adverse cardiovascular events; HR, hazard ratio; CI, confidence interval. The significance was tested by univariate and multivariate Cox regression analyses.

^*^*p*<0.05; ^**^*p*<0.001.

**Supplemental Table 17. Sensitivity analyses of Lp(a) for predicting MACEs in the confirmatory cohort by including AB group**

| **Lp(a) categories (Events/Total subjects)** | **Crude Model**  **HR (95% CI)** | **Adjusted Model**  **HR (95% CI)** |
| --- | --- | --- |
| Low Lp(a) (67/2349) | 1.00 (reference) | 1.00 (reference) |
| Medium Lp(a) (74/2000) | 1.32 (0.95-1.84) | 1.20 (0.86-1.67) |
| High Lp(a) (84/1927) | 1.61 (1.17-2.22)^*^ | 1.38 (0.99-1.92) |
| Per 1-SD increase of LgLp(a) | 1.22 (1.09-1.37)^*^ | 1.17 (1.02-1.31)^*^ |

Total non-O participants = 6276. Adjusted model adjusted for age, sex, body mass index, hypertension, diabetes mellitus, current smoking, prior myocardial infarction, prior revascularization, low-density lipoprotein cholesterol, triglyceride, high-sensitivity C-reactive protein, creatinine, left ventricular ejection fraction, and statin use. Lp(a), lipoprotein(a); MACEs, major adverse cardiovascular events; HR, hazard ratio; CI, confidence interval. The significance was tested by univariate and multivariate Cox regression analyses.

^*^*p*<0.05; ^**^*p*<0.001.

**Supplemental Table 18. Sensitivity analyses of ABO blood group and Lp(a) for predicting MACEs in the exploratory cohort by including AB group**

| **Category (Events/Total subjects)** | **Crude Model**  **HR (95% CI)** | **Adjusted Model**  **HR (95% CI)** |
| --- | --- | --- |
| ABO blood group |  |  |
| O group (151/2453) | 1.00 (reference) | 1.00 (reference) |
| A group (188/2338) | 1.36 (1.04-1.78)^*^ | 1.36 (1.02-1.81)^*^ |
| B group (221/2820) | 1.33 (1.02-1.73)^*^ | 1.37 (1.04-1.81)^*^ |
| AB group (50/877) | 1.05 (0.71-1.54) | 0.94 (0.63-1.42) |
| Lp(a) |  |  |
| Low Lp(a) (161/3166) | 1.00 (reference) | 1.00 (reference) |
| Medium Lp(a) (226/2797) | 1.55 (1.20-2.00)^**^ | 1.51 (1.15-1.98)^*^ |
| High Lp(a) (223/2525) | 1.71 (1.32-2.21)^**^ | 1.71 (1.31-2.25)^**^ |
| Per 1-SD increase of LgLp(a) | 1.23 (1.11-1.37)^*^ | 1.22 (1.09-1.36)^**^ |
| ABO blood group and Lp(a) |  |  |
| O group-Low Lp(a) (44/925) | 1.00 (reference) | 1.00 (reference) |
| O group-Medium Lp(a) (50/764) | 1.34 (0.79-2.27) | 1.18 (0.68-2.08) |
| O group-High Lp(a) (57/764) | 1.70 (1.03-2.81)^*^ | 1.63 (0.97-2.74) |
| Non-O group-Low Lp(a) (117/2241) | 1.19 (0.76-1.86) | 1.13 (0.71-1.79) |
| Non-O group-Medium Lp(a) (176/2033) | 1.91 (1.25-2.92)^*^ | 1.79 (1.15-2.78)^*^ |
| Non-O group-High Lp(a) (166/1761) | 2.04 (1.33-3.14)^*^ | 1.96 (1.25-3.06)^*^ |

Total participants = 8488. Adjusted model adjusted for sex, age, body mass index, hypertension, diabetes mellitus, current smoking, prior myocardial infarction, prior revascularization, low-density lipoprotein cholesterol, triglyceride, high-sensitivity C-reactive protein, creatinine, left ventricular ejection fraction, statin use, ABO blood groups, and Lp(a), other than the variables being analyzed. Lp(a), lipoprotein(a); LgLp(a), log-transformed Lp(a); MACEs, major adverse cardiovascular events; HR, hazard ratio; CI, confidence interval. The significance was tested by univariate and multivariate Cox regression analyses.

^*^*p*<0.05; ^**^*p*<0.001.

**Supplemental Table 19. Sensitivity analyses of ABO blood group and Lp(a) for predicting MACEs in the confirmatory cohort by including AB group**

| **Category (Events/Total subjects)** | **Crude Model**  **HR (95% CI)** | **Adjusted Model**  **HR (95% CI)** |
| --- | --- | --- |
| ABO blood group |  |  |
| O group (62/2514) | 1.00 (reference) | 1.00 (reference) |
| A group (93/2456) | 1.54 (1.12-2.13)^*^ | 1.55 (1.12-2.14)^*^ |
| B group (99/2946) | 1.37 (1.00-1.88) | 1.37 (1.00-1.88) |
| AB group (33/874) | 1.59 (1.04-2.42)^*^ | 1.57 (1.03-2.40)^*^ |
| Lp(a) |  |  |
| Low Lp(a) (85/3298) | 1.00 (reference) | 1.00 (reference) |
| Medium Lp(a) (93/2768) | 1.33 (0.99-1.78) | 1.20 (0.89-1.62) |
| High Lp(a) (109/2724) | 1.63 (1.23-2.17)^**^ | 1.44 (1.08-1.93)^*^ |
| Per 1-SD increase of LgLp(a) | 1.18 (1.06-1.31)^*^ | 1.21(1.06-1.38)^*^ |
| ABO blood group and Lp(a) |  |  |
| O group-Low Lp(a) (18/949) | 1.00 (reference) | 1.00 (reference) |
| O group-Medium Lp(a) (19/768) | 1.33 (0.70-2.54) | 1.24 (0.65-2.36) |
| O group-High Lp(a) (25/797) | 1.74 (0.95-3.18) | 1.68 (0.92-3.10) |
| Non-O group-Low Lp(a) (67/2349) | 1.52 (0.90-2.56) | 1.60 (0.95-2.69) |
| Non-O group-Medium Lp(a) (74/2000) | 2.01 (1.20-3.36)^*^ | 1.92 (1.14-3.23)^*^ |
| Non-O group-High Lp(a) (84/1927) | 2.45 (1.47-4.07)^**^ | 2.21 (1.32-3.69)^*^ |

Total participants = 8790. Adjusted model adjusted for age, sex, body mass index, hypertension, diabetes mellitus, current smoking, prior myocardial infarction, prior revascularization, low-density lipoprotein cholesterol, triglyceride, high-sensitivity C-reactive protein, creatinine, left ventricular ejection fraction, statin use, ABO blood group, and Lp(a), other than the variables being analyzed. Lp(a), lipoprotein(a); LgLp(a), log-transformed Lp(a); MACEs, major adverse cardiovascular events; HR, hazard ratio; CI, confidence interval. The significance was tested by univariate and multivariate Cox regression analyses.

^*^*p*<0.05; ^**^*p*<0.001.


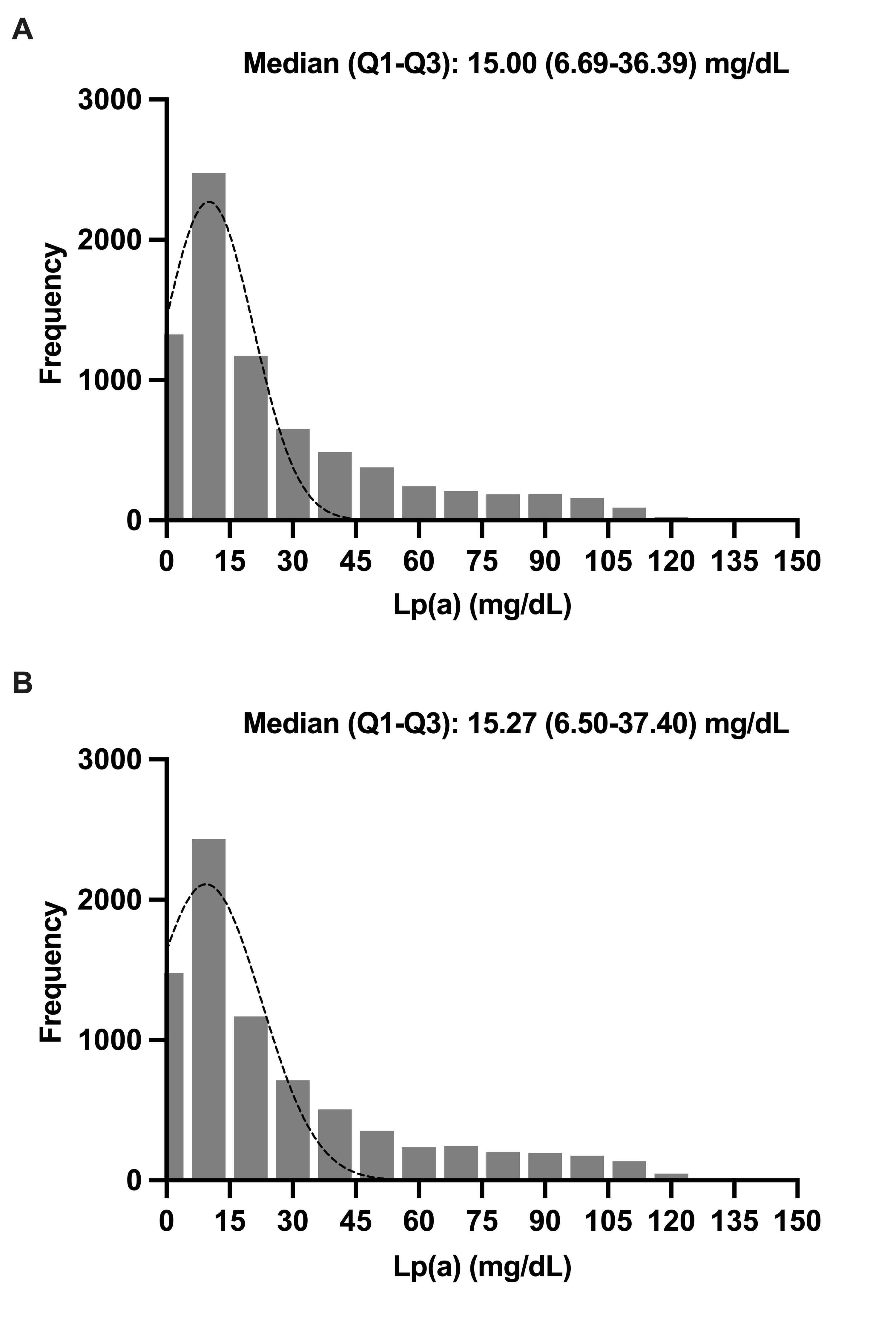


**Supplemental Figure 1. The distribution of plasma Lp(a) concentrations in the (A) exploratory (n=7611) and (B) confirmatory (n=7916) cohorts** Lp(a), lipoprotein(a); Q, quartile.


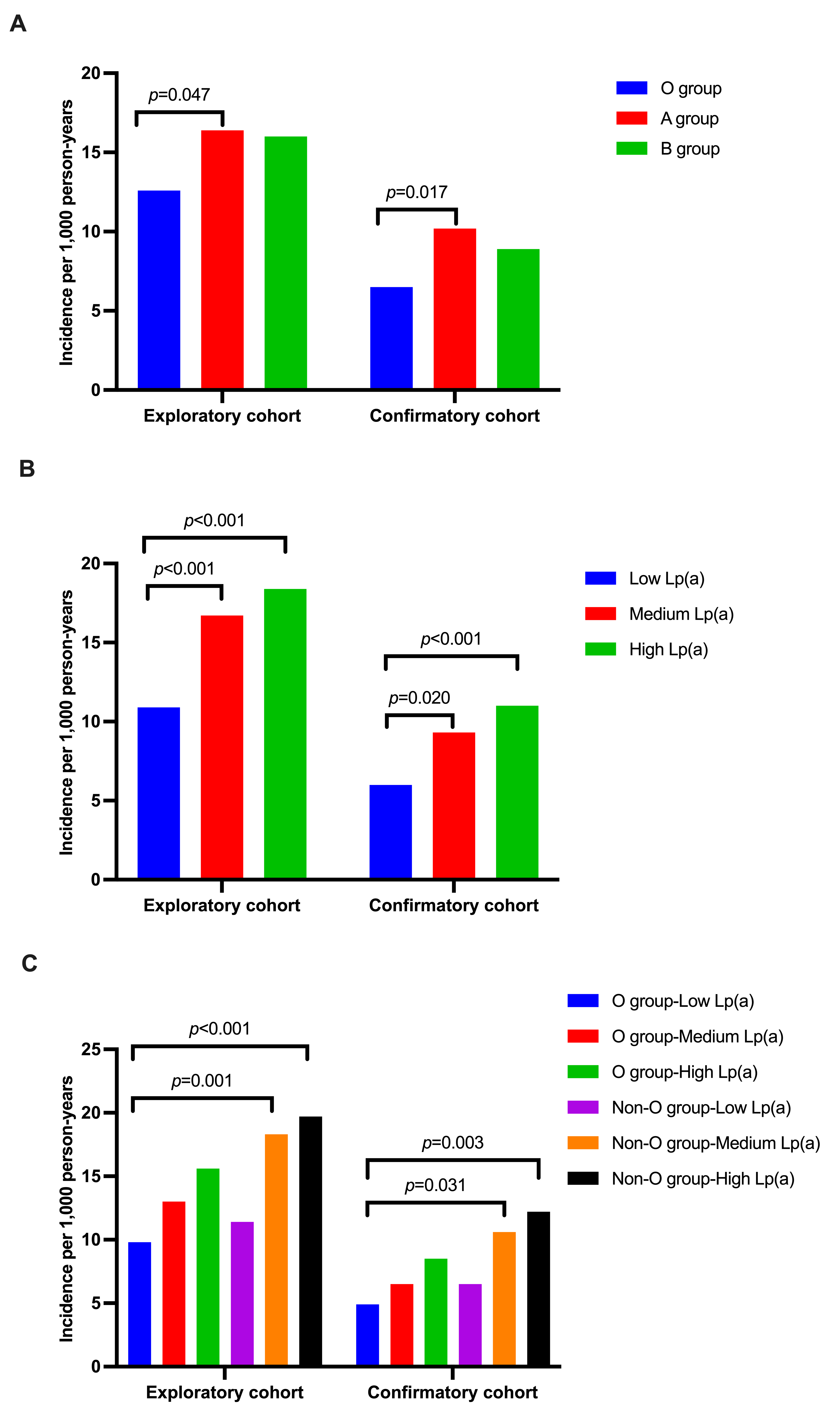


**Supplemental Figure 2. The comparison of incidence of MACEs according to ABO blood group and/or Lp(a) in exploratory cohort (n=7611) and confirmatory cohort (n=7916)** (A) according to ABO blood group†; (B) according to Lp(a) levels; (C) according to both ABO blood group† and Lp(a) levels. Lp(a), lipoprotein(a); MACEs, major adverse cardiovascular events. The chi square test with Bonferroni corrections was applied to compare the differences between groups. Adjusted *p*-values were reported. †Excluded AB blood group due to its small sample size.

**
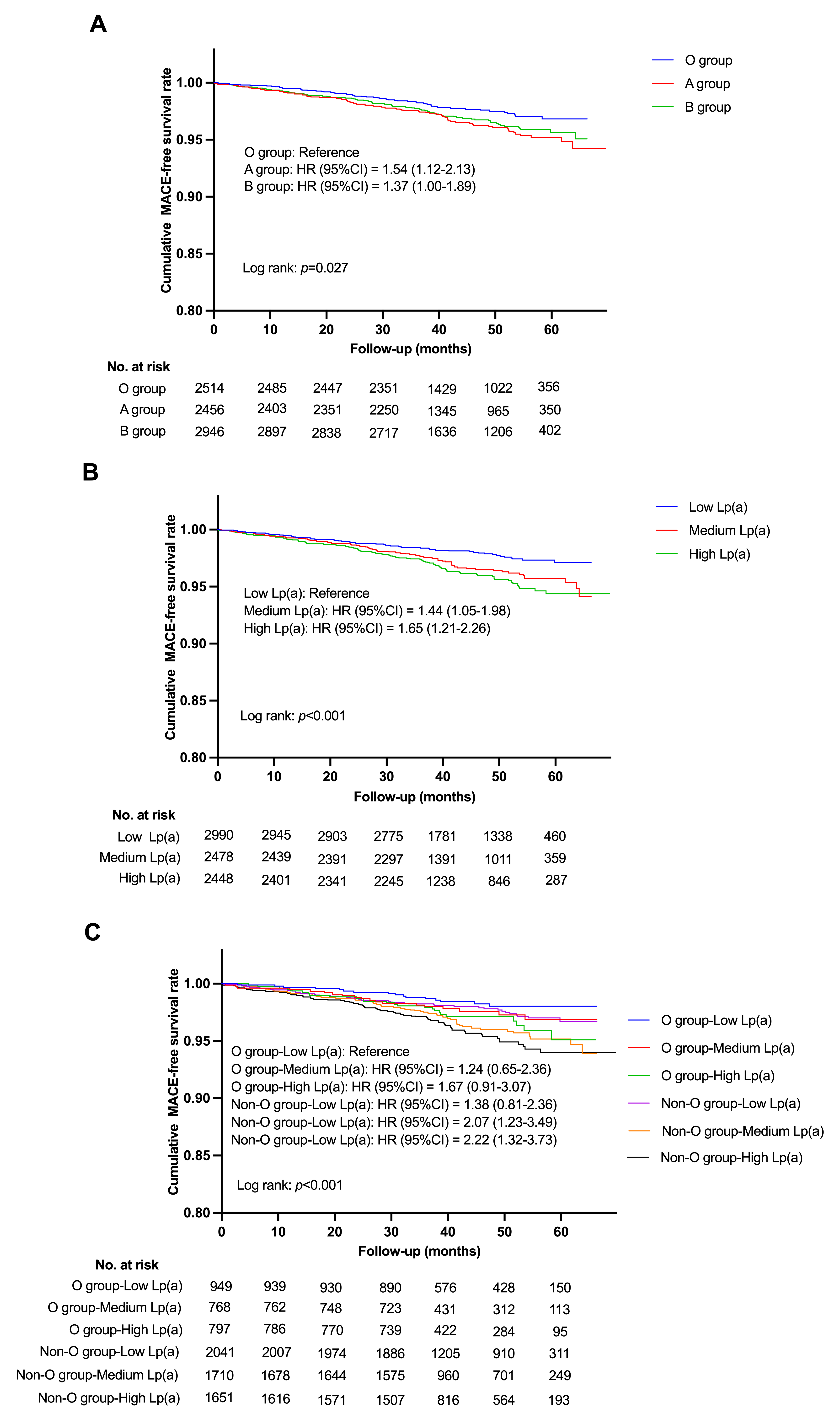
**

**Supplemental Figure 3. Cumulative MACE-free survival rate according to ABO blood group and/or Lp(a) levels in the confirmatory cohort** (A) ABO blood group†; (B) Lp(a) levels; (C) Both ABO blood group† and Lp(a) levels. (MACEs = 254, Total participants = 7916). HR, hazard ratio; Lp(a), lipoprotein(a); MACEs, major adverse cardiovascular events (composite of cardiovascular death, non-fatal myocardial infarction, or ischemic stroke). The significance was examined by the log-rank tests. HRs were based on Cox regression analyses adjusted for sex, age, body mass index, hypertension, diabetes mellitus, current smoking, prior myocardial infarction, prior revascularization, low-density lipoprotein cholesterol, triglyceride, high-sensitivity C-reactive protein, creatinine, left ventricular ejection fraction, statin use, ABO blood groups, and Lp(a), other than the variables being analyzed. †Excluded AB blood group due to its small sample size.


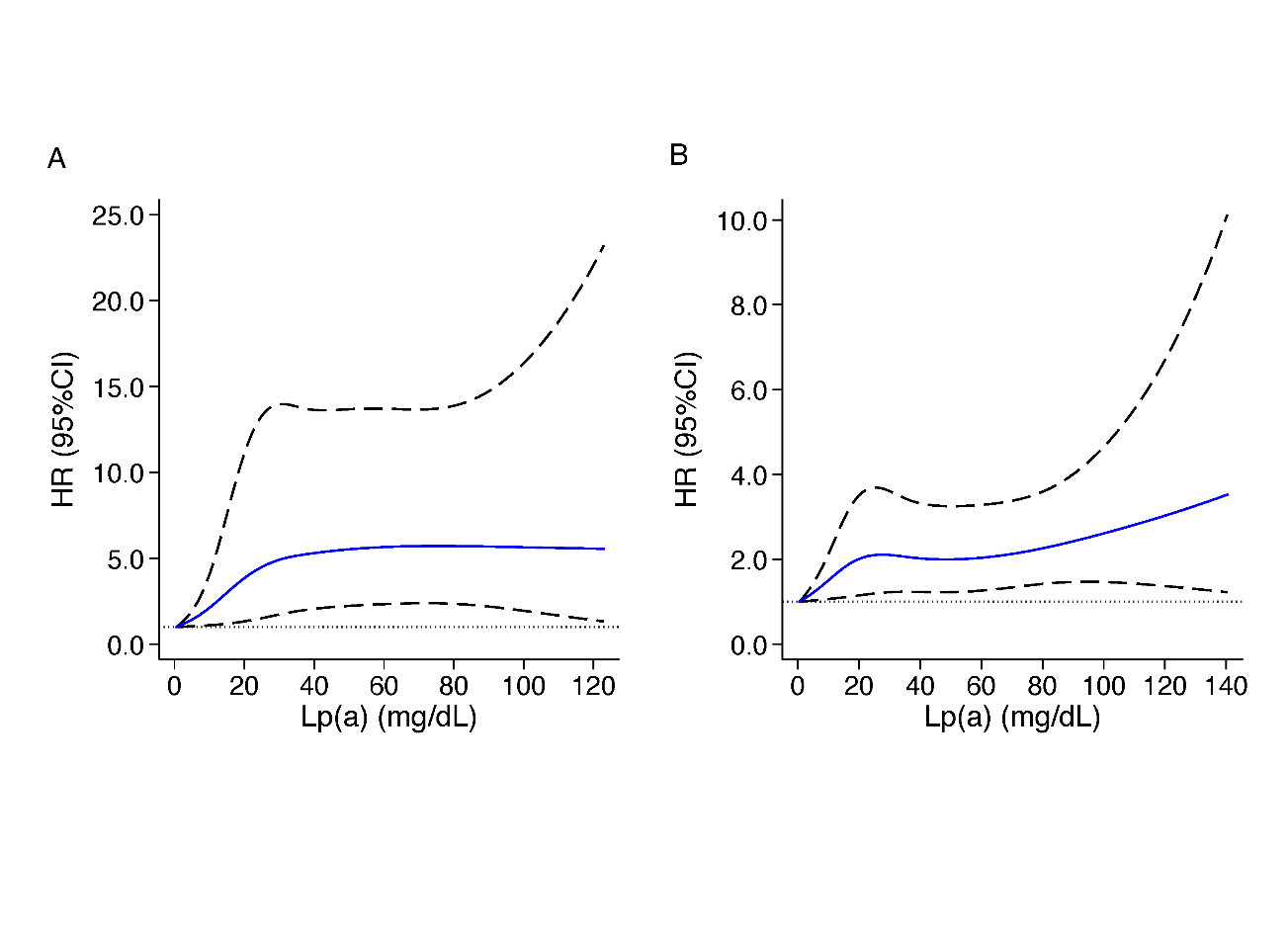


**Supplemental Figure 4. Sex- and age- adjusted RCS plot** **of Lp(a) and risk of MACEs according to ABO blood groups among patients in the confirmatory cohort** (A) O blood group; (B) A or B blood group. Events = 254, Total participants = 7916. HR, hazard ratio; Lp(a), lipoprotein(a); RCS, restricted cubic spline; MACEs, major adverse cardiovascular events.


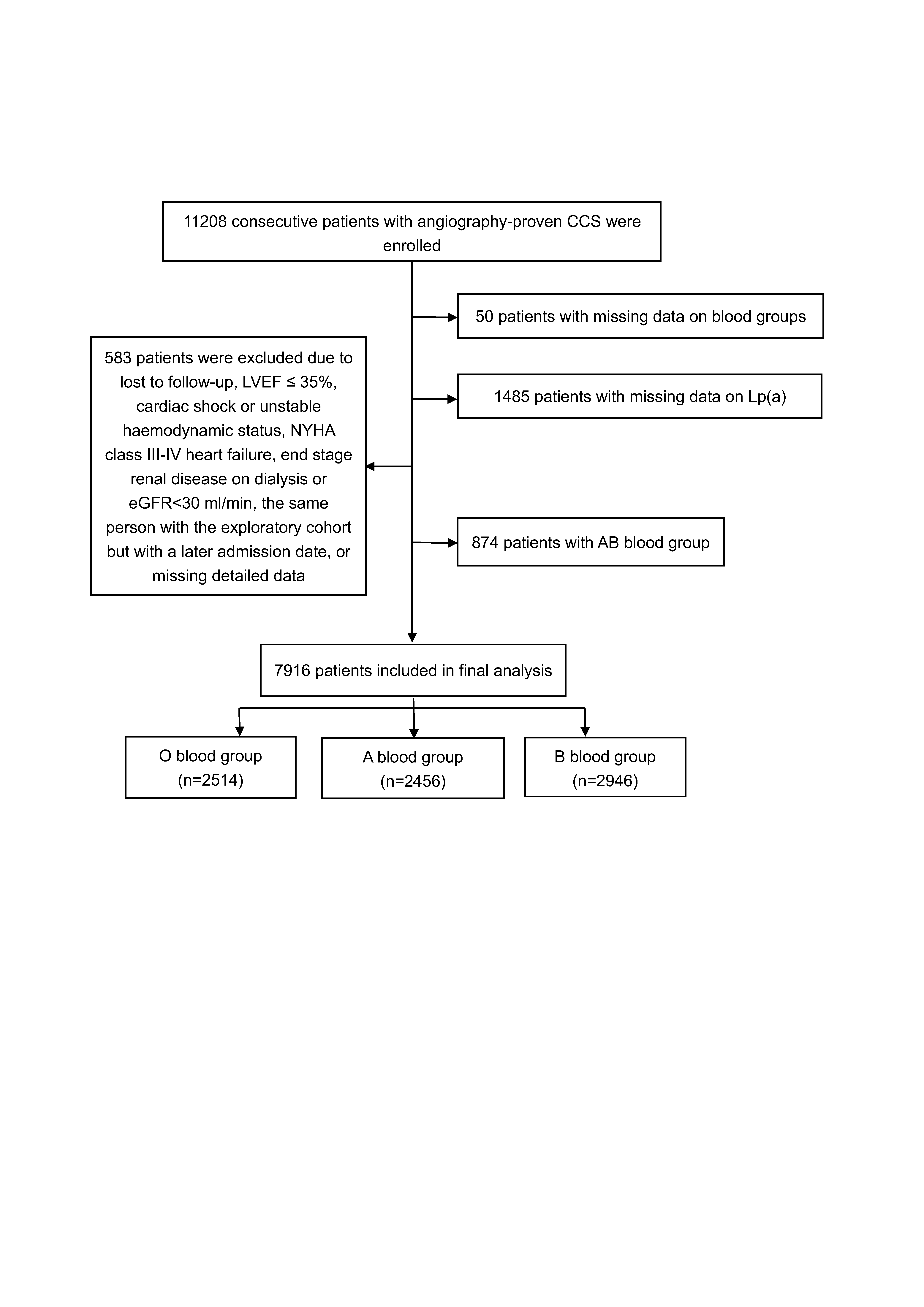


**Supplemental Figure 5. The flowchart illustrating the confirmatory cohort (n=7916)** CCS, chronic coronary syndrome; Lp(a), lipoprotein(a); LVEF, left ventricular ejection fraction; NYHA, New York Heart Association; eGFR, estimated glomerular filtration rate.
